# A redox signalling globin is essential for reproduction in *Caenorhabditis elegans*

**DOI:** 10.1038/ncomms9782

**Published:** 2015-12-01

**Authors:** Sasha De Henau, Lesley Tilleman, Matthew Vangheel, Evi Luyckx, Stanislav Trashin, Martje Pauwels, Francesca Germani, Caroline Vlaeminck, Jacques R. Vanfleteren, Wim Bert, Alessandra Pesce, Marco Nardini, Martino Bolognesi, Karolien De Wael, Luc Moens, Sylvia Dewilde, Bart P. Braeckman

**Affiliations:** 1 Department of Biology, Ghent University, Ghent B-9000, Belgium; 2 Department of Biomedical Sciences, University of Antwerp, Antwerp B-2000, Belgium; 3 Department of Chemistry, University of Antwerp, Antwerp B-2000, Belgium; 4 Department of Physics, University of Genova, Genova I-16146, Italy; 5 Department of Biosciences, University of Milano, Milano I-20133, Italy; 6 CNR-IBF and CIMAINA, University of Milano, Milano I-20133, Italy

## Abstract

Moderate levels of reactive oxygen species (ROS) are now recognized as redox signalling molecules. However, thus far, only mitochondria and NADPH oxidases have been identified as cellular sources of ROS in signalling. Here we identify a globin (GLB-12) that produces superoxide, a type of ROS, which serves as an essential signal for reproduction in *C. elegans*. We find that GLB-12 has an important role in the regulation of multiple aspects in germline development, including germ cell apoptosis. We further describe how GLB-12 displays specific molecular, biochemical and structural properties that allow this globin to act as a superoxide generator. In addition, both an intra- and extracellular superoxide dismutase act as key partners of GLB-12 to create a transmembrane redox signal. Our results show that a globin can function as a driving factor in redox signalling, and how this signal is regulated at the subcellular level by multiple control layers.

## Abstract (web summary)

Globins are best known for their role in respiration, but recent studies suggest they might contribute to redox signalling as well. Here, the authors present biochemical, structural and *in vivo* evidence that the roundworm globin Glb-12 acts as a superoxide generator necessary for germline development.

## Graphical abstract

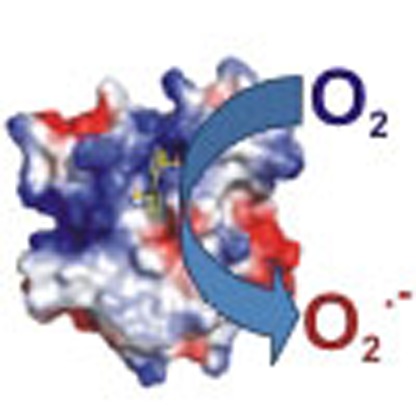

Reactive oxygen species (ROS)-based redox signalling is involved at all levels of cellular organization, from cell differentiation to cell death^1^,^2^. In this type of signalling, low levels of ROS act as biological messengers by inducing reversible oxidative modifications in downstream proteins and thereby influencing signalling pathways^3^,^4^. These ROS are enzymatically generated by cells to serve this signalling function. However, in the majority of the reported redox-sensitive signalling pathways, it is often not known which proteins are responsible for generating the redox signal and how cells spatially and temporally link ROS production to specific signalling pathways and so achieve desired cellular outcomes.

Globins are increasingly hypothesized to play a role in redox biology^5^. These proteins are characterized by a common tertiary structure and the presence of a haem group, whereby their function is largely determined by how this haem group is incorporated in the surrounding globin fold. The best-characterized function of globins is in oxygen diffusion and transport, as exemplified by vertebrate haemoglobin and myoglobin. The haem iron in these globins is pentacoordinated, leaving the sixth coordination site of the iron free for reversible binding of diatomic ligands, such as O_2_. In a second type of globins, named hexacoordinated globins, all six positions of the haem iron are bound^6^. Since their initial discovery two decades ago, these hexacoordinated globins are now recognized to be broadly present in plants and animals^7^. Because of the hexacoordinated nature of the haem iron, ligand binding/transfer becomes more complex^8^ or even absent^9^. On the other hand, this hexacoordinated state of the iron seems to favour electron transfer^9^,^10^,^11^, indicating that redox reactions involving the haem iron could be a key element in the physiological function of these globins. Therefore, increasing attention has been placed on the potential roles of hexacoordinated globins and globin-like proteins in signalling and redox chemistry, such as protection against ROS and electron transfer to molecular partners^7^. However, even though a fairly detailed understanding on the structure and biochemistry of hexacoordinated globins is now present, it has thus far been extremely difficult to understand what their possible physiological roles are.

*C. elegans* has thirty-three globin-like proteins, which display different sizes, are differently located and are likely to be functionally diverse^12^. This makes *C. elegans* an attractive model to study the potential roles of globins and globin-like proteins in redox metabolism. A genome-wide RNAi screen in *C. elegans* reported abnormal egg laying and embryonic lethality following RNAi for GLB-12 (ref. 13), providing a clear phenotype for this so far uncharacterized globin. In this study, we analyse the role of GLB-12 in more detail and show that this protein is hexacoordinated, functions as a redox signalling protein and has an essential role in the reproduction of *C. elegans*.

## Results

### GLB-12 is essential for reproduction

**Figure 1:**
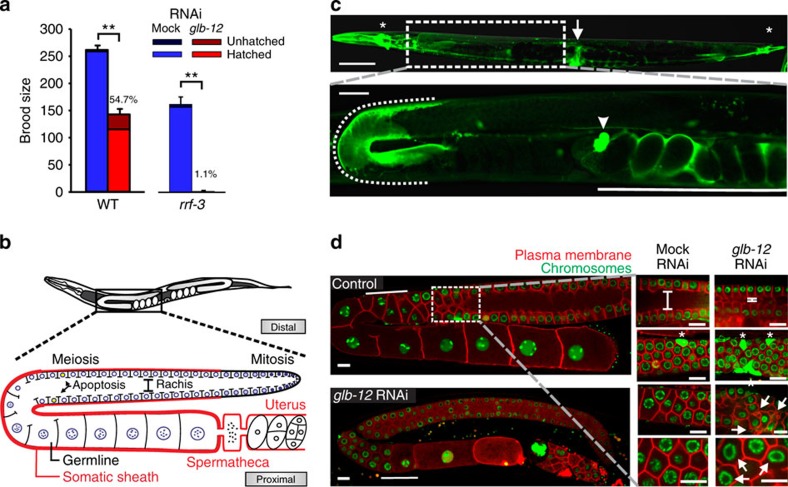
GLB-12 is essential for reproduction. (**a**) *glb-12* RNAi in the WT (*n*=16 biological replicates) and RNAi-hypersensitive strain *rrf-3* (*n*=3) caused a decreased fecundity. **P<0.01 (two-sided Student *t*-test); data are represented as mean±s.e.m. (**b**) Schematic representation of the *C. elegans*' reproductive system anatomy. (**c**) GLB-12 is expressed in neurons (asterisk), the vulva (arrow) (upper panel, scale bar: 100 μm), and very specific regions of the somatic gonad (lower panel, scale bar, 20 μm), that is, the somatic sheath (dotted line), the proximal part of the spermatheca (arrowhead) and the uterus (full line). (**d**) *glb-12* RNAi caused multiple defects in the germline. Solid line indicates the meiotic transition region pachytene—diplotene—diakinesis (left panels); bar shows the rachis size; asterisks indicate apoptotic cells and arrows indicate irregular compartment junctions and nuclei (right panels). Scale bar: 10 μm.

To characterize the role of GLB-12 in reproduction, we analysed the effect of reduced GLB-12 levels on the brood size of *C. elegans*. We observed that *glb-12* RNAi reduced fecundity and increased embryonic lethality in wild-type (WT) worms and caused sterility in the majority of worms in the RNAi-hypersensitive strain *rrf-3* (Fig. 1a). Expression analysis showed that GLB-12 is present in distinct parts of the somatic reproductive system (Fig. 1b,c; Supplementary Fig. 1a)—the distal gonadal sheath, the proximal part of the spermatheca, and the uterus—and in several head and tail neurons and the vulva. GLB-12 does not appear to be directly present in the germline: integration of the GLB-12::GFP reporter, which is necessary to allow expression in the germline, showed no visible expression in this tissue (Supplementary Fig. 1c). Further, GLB-12 appears to act directly from the somatic gonad to regulate reproduction: *glb-12* RNAi only had an effect on reproduction in a soma-specific, but not in germline- or neuronal-specific RNAi strains (Supplementary Fig. 1b), while *glb-12* RNAi in the GLB-12 reporter strain reduced reporter levels in the somatic gonad only (Supplementary Fig. 1c).

To understand the impact of GLB-12 on gonad morphology, we used a strain visualizing the germline architecture by fluorescent markers (Fig. 1b,d). In the first generation, *glb-12* RNAi caused a range of defects in the adult germline (Fig. 1d): delayed meiotic progression, a considerably smaller rachis, increased apoptosis levels, and irregular shaped compartments and nuclei. In the second generation, *glb-12* RNAi led to abnormal germline development and failure to produce oocytes (Supplementary Fig. 1d). GLB-12 is thus essential for germline development and regulation and appears to be involved in several aspects of reproduction.

### GLB-12 is a hexacoordinated globin with redox properties

**Figure 2:**
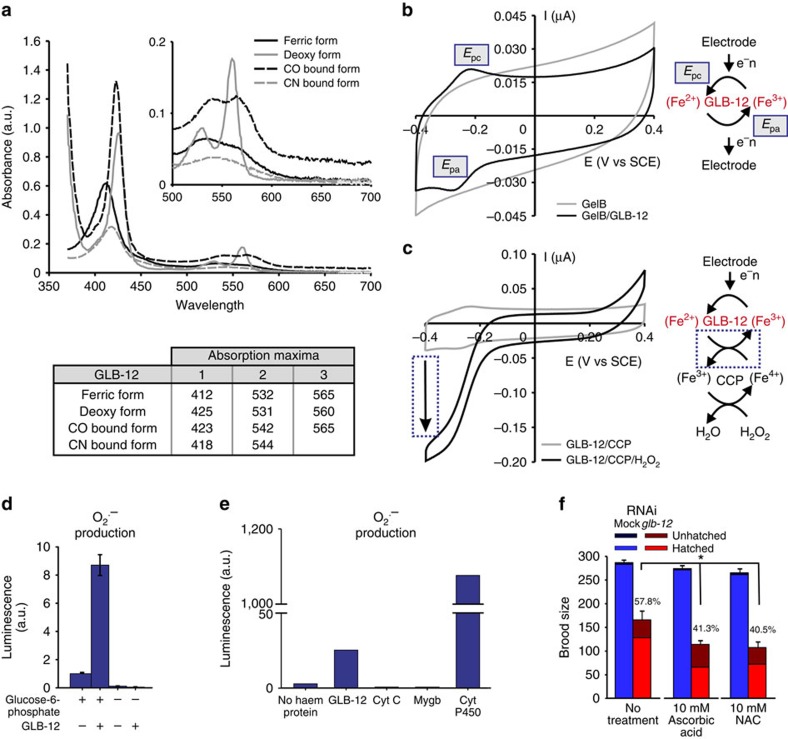
GLB-12 is a hexacoordinated globin with redox properties and is capable of producing O_2_^.−^. (**a**) UV–vis spectra of different GLB-12 forms, with their absorption maxima. The absorption spectrum of purified GLB-12 showed that it is spontaneously oxidized to the ferric state (Fe^3+^) upon exposure to air. Reducing GLB-12 (Fe^2+^) resulted in an absorption spectrum that is typical for a hexacoordinated haem iron. Absorption spectra of CN- and CO-bound GLB-12 could be obtained after exposure of GLB-12 to an excess of KCN or CO. (**b**) The current potential behaviour of a gelatin B (GelB) and a GelB/GLB-12 electrode, with the reduction (*E*_pc_) and oxidation peak (*E*_pa_), showing that GLB-12 is reversibly oxidized and reduced with a reduction potential of −0.244 V (versus SCE). The reversible oxidation/reduction of GLB-12 by cyclic voltammetry is illustrated in the scheme. (**c**) The current potential behaviour of a gelatin B electrode containing GLB-12 and CCP (cytochrome C peroxidase), with and without H_2_O_2_. The arrow points to the increase of the reduction peak when H_2_O_2_ is added, showing that GLB-12 becomes fully reduced by electron transfer to CCP, making CCP capable of reducing H_2_O_2_. This reaction is illustrated in the scheme. (**d**) GLB-12 is capable of producing O_2_^.−^. (+) indicates the presence, (−) the absence of glucose-6-phosphate and GLB-12. (**e**) Comparison of *in vitro* superoxide production by GLB-12, cytochrome C, myoglobin and cytochrome P450 (Cyt P450). Myoglobin and cytochrome c, which have a reduction potential higher than the O_2_/O_2_^.−^ couple^64^,^65^ and therefore should not be able to produce O_2_^.−^, did not lead to an increase in luminescence. Cytochrome P450, which has a reduction potential lower than the O_2_/O_2_^.−^ couple and can produce high levels of O_2_^.−^ (ref. 66), led to a large increase in luminescence. GLB-12 led to a moderate increase in luminescence, indicating that it produced moderate levels of O_2_^.−^. (**f**) The antioxidants ascorbic acid and *N*-acetylcysteine (NAC) enhance the *glb-12* RNAi reduction in fecundity (*n*=4). Percentages show control RNAi compared with *glb-12* RNAi. Data are represented as mean±s.e.m.; *P<0.05 (two-sided Student *t*-test).

To understand how GLB-12 could influence reproduction on a molecular level, we analysed the biochemical and structural properties of the purified globin. Spectroscopic analysis, which allows to discriminate between different globin forms, showed that reduced deoxy (Fe^2+^) GLB-12 displays a hexacoordinated haem iron (Fig. 2a; Supplementary Fig. 2a). Hexacoordinated globins are present in prokaryotes, plants and animals, but their function is largely unclear^7^. They can potentially bind diatomic ligands such as O_2_, CN^−^ and CO to their haem iron, thereby replacing the endogenous HisE7 ligand. However, even though GLB-12 was able to bind CN^−^ and CO *in vitro* (Fig. 2a), both reactions proceeded very slowly, requiring several minutes (reaction kinetics not shown). In addition, upon exposure to air, GLB-12 is spontaneously oxidized to the ferric state (Fe^3+^) (Fig. 2a) and is thus incapable of binding O_2_ under physiological conditions. These results argue against a role for GLB-12 in reversible ligand binding and/or transport, the best known function for globins. On the other hand, the spontaneous oxidation of air-exposed GLB-12 is in line with the observation that hexacoordination appears to lower the redox potential and promotes electron transfer of the haem iron^9^,^10^,^11^. By using cyclic voltammetry to study the electrochemical properties of GLB-12 in more detail, we indeed observed a relatively low reduction potential for the GLB-12 redox couple Fe^2+^/Fe^3+^ (−0.244 V versus saturated calomel reference electrode (SCE)), which is largely stable within the physiological pH range (Fig. 2b; Supplementary Fig. 2b). To determine whether GLB-12 could transfer electrons to other molecules, we included cytochrome c peroxidase (CCP) in this experimental setup as an electron acceptor and subsequent catalyst for H_2_O_2_ reduction. It is important to note that CCP by itself shows no electrocatalytic activity under these conditions^14^. When H_2_O_2_ was added to GLB-12 and CCP, the reduction current increased and the oxidation peak disappeared, indicating very fast GLB-12 oxidation (Fig. 2c). This shows that GLB-12 can donate electrons to CCP in a continuous manner, allowing the latter to reduce H_2_O_2_. Compared to other tested globins, GLB-12 showed a 10-fold higher electron transfer rate to CCP (Supplementary Fig. 2c). Combined, these results support a role for GLB-12 in electron transfer reactions.

### GLB-12 produces O_2_^.−^ as a signalling molecule

Because GLB-12 can participate in electron transfer and has a reduction potential lower than the O_2_/O_2_^.−^ couple^15^, it may directly interact with O_2_ to generate O_2_^.−^. To test this hypothesis, we used an *in vitro* enzymatic reduction system for haem proteins^16^ and included lucigenin, which emits light upon reaction with O_2_^.−^ (Supplementary Fig. 2d). Addition of GLB-12 and glucose-6-phosphate, the driving force of this system, led to increased luminescence, showing that GLB-12 can convert O_2_ to O_2_^.−^ (Fig. 2d; Supplementary Fig. 2d). O_2_^.−^ levels are proportional to GLB-12 concentration, indicative of a 1:1 stoichiometry (Supplementary Fig. 2e). We also validated this *in vitro* method using haem proteins with a reduction potential higher and lower than the O_2_/O_2_^.−^ couple (Fig. 2e). Noteworthy, O_2_^.−^ production rate of cytochrome P450 was 40-fold higher than that of GLB-12. We subsequently hypothesized that *in vivo* O_2_^.−^ produced by GLB-12 could act as a redox signalling molecule. Decreasing GLB-12 levels by RNAi would thus decrease this O_2_^.−^ signal, and exposing worms to O_2_^.−^ scavengers could potentially even further reduce the O_2_^.−^ signal. Using the antioxidants *N*-acetylcysteine (NAC) or ascorbic acid, scavengers of O_2_^.−^ and other ROS, indeed further aggravated the reduction in brood size by *glb-12* RNAi (Fig. 2f), providing a first indication that GLB-12 generates O_2_^.−^ as a signalling molecule.

**Figure 3:**
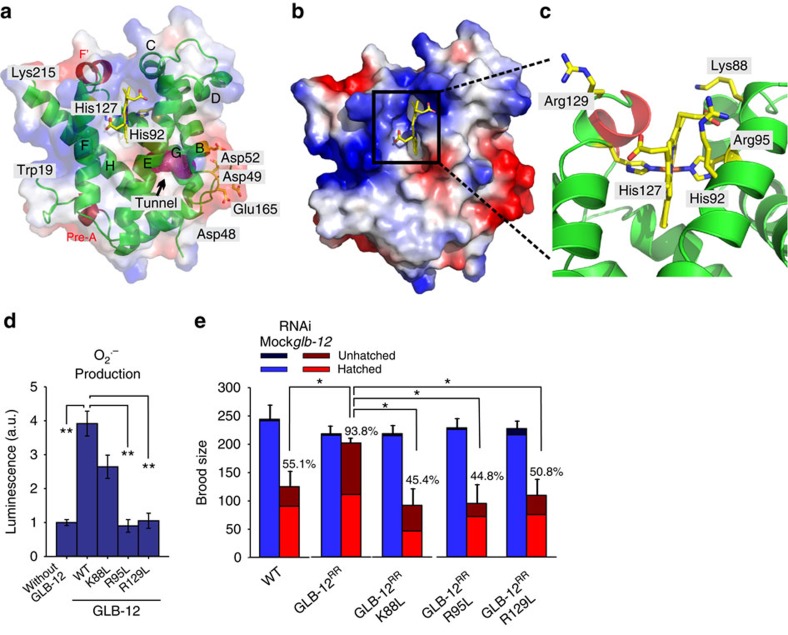
GLB-12 produces O_2_^.−^ as a signalling molecule. (**a**) Ribbon representation of the GLB-12 globin domain, with helices labelled according to the canonical globin fold (green). The GLB-12-specific pre-A helix and F'-helix are highlighted in red. The terminal residues visible in the electron density (19–215) are labelled. The protein electrostatic surface (see also panel (**b**)) is displayed in semitransparent colours, allowing the view of underlying secondary structure elements and a distal apolar tunnel (magenta mesh) located between the B-, E- and G-helices. Acidic residues, localized where this apolar tunnel reaches the protein surface, are shown in stick representation (yellow) and labelled. (**b**) Electrostatic surface of the GLB-12 globin domain. The blue and red colours highlight positively and negatively charged surfaces, respectively. The haem moiety, with the propionate groups fully exposed to the solvent region, is shown as stick representation (yellow). (**c**) Detail of the GLB-12 haem cavity, showing the presence of two histidines and three additional polar amino acids. (**d**) The GLB-12 K88L mutant is less capable of O_2_^.−^ production, while R95L and R129L mutants are incapable of O_2_^.−^ production. (**e**) These three mutants, when expressed as RNAi-resistant (RR) genes, are not capable of rescuing the *glb-12* RNAi reduction in fecundity (*n*=5). Percentages show control RNAi compared with *glb-12* RNAi within a strain. *P<0.05; **P<0.01 (two-sided Student *t*-test). Data are mean±s.e.m.

The crystal structure of the GLB-12 globin domain, refined to 1.65 Å resolution (Table 1), further supported our current findings. It confirmed haem hexacoordination, with strong Fe-N bonds with the distal His92 and the proximal His127 residues (Fig. 3a; Supplementary Fig. 3a). Overall, the hexacoordinated GLB-12 globin domain is closely related to the 3D structures of human and murine neuroglobin^10^,^17^,^18^,^19^, human cytoglobin^20^, non-symbiotic rice haemoglobin^21^, *C. elegans* GLB-6 (ref. 9) and *Geobacter sulfurreducens* globin-coupled sensor^22^, although GLB-12 possesses several unique structural features. The proximal His127 is highly exposed to the solvent, which is the result of a hydrogen bond between the A-propionate of the haem to Ser126 OH. As a result, two water molecules are hosted at the entrance of the proximal site (hydrogen bonded to Ser126 OG and to Gln130 NE2, respectively), and four additional water molecules and one acetate ion fall next to the propionate (Supplementary Fig. 3a). This exposure to a polar aqueous environment would favour oxidation of the ferrous haem, which is consistent with the low GLB-12 redox potential reported. On the distal site of the haem cavity, the D-propionate stabilizes the location of the E-helix N-terminal region through hydrogen bonding (Supplementary Fig. 3a). In addition, residues Phe89 and Phe96 (surrounding the distal His92) are part of a wide cluster of aromatic/hydrophobic residues (Val54, Phe58, Leu59, Val62, Phe73, Phe152) (not highlighted). These could restrict E-helix movements required to achieve a pentacoordinated haem and would thereby limit ligand binding. This explains the slow CN^−^ and CO binding we observed. Additionally, this aromatic/hydrophobic residue cluster creates a tunnel of about 50 Å^3^ in the globin domain, nestled among the B-, E-, and G-helices (Fig. 3a). Interestingly, this otherwise apolar tunnel shows a strong negative charge distribution at the solvent exit (resulting from residues Asp48, Asp49, Asp52 and Glu165) (Fig. 3a,b). In the crystal structure the tunnel exit to the solvent appears rather narrow; however, structural fluctuations may vary its diameter, providing a direct connection between the haem distal site and the solvent region. Given this, it is possible that the negative charges located at the tunnel exit would help to remove small and negatively charged species such as O_2_^.−^, produced by redox reactions at the distal site, from the protein core. Further, while the haem pocket in globins usually prevents haem iron oxidation by creating a hydrophobic environment, in GLB-12 three polar amino acids (Lys88, Arg95 and Arg129) are located at the edge of the haem cavity (Fig. 3c), possibly to enhance solvent access to the haem and stimulate haem iron oxidation and O_2_^.−^ production. Mutation of these three polar amino acids to the hydrophobic leucine indeed reduced or prevented O_2_^.−^ production (Fig. 3d), while other biochemical characteristics remained largely unaffected (Supplementary Fig. 3b–d). Furthermore, transgene animals bearing these mutations in an RNAi-resistant *glb-12* gene (Supplementary Fig. 3e,f) failed to show rescue of the reduced brood size following *glb-12* RNAi (Fig. 3e). Also the K88L mutant, even though it can still produce some O_2_^.−^, failed to rescue the reduction in brood size. Overall, these results show that GLB-12 possesses specific structural properties associated with a role in redox biology and further support that GLB-12 produces O_2_^.−^ as a signalling molecule.

### GLB-12 shows a distinct tissue and subcellular expression

**Figure 4:**
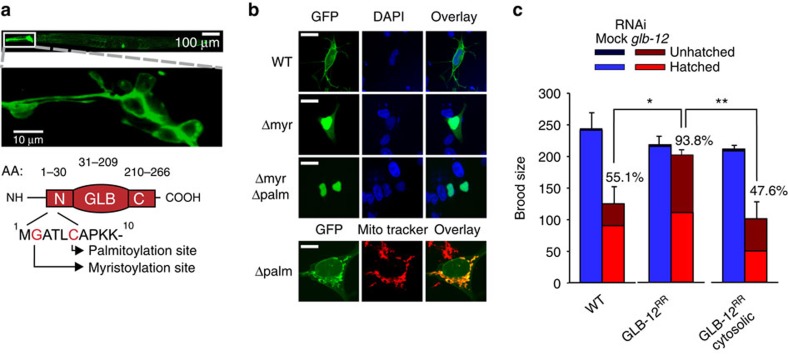
GLB-12 shows a distinct tissue and subcellular expression pattern. (**a**) GLB-12 is membrane-bound, as seen in head neurons. Upper scale bar: 100 μm; lower scale bar, 10 μm. The graphic shows the presence of a myristoylation and palmitoylation site in the N-terminal region (N: N-terminal, C: C-terminal, AA: amino acid). (**b**) Deletion of the myristoylation or palmitoylation site disrupts cell membrane localization, as seen in human neuroblastoma SH-SY5Y cells. Scale bar: 10 μm. (**c**) A cytosolic RNAi-resistant (RR) GLB-12 is not capable of rescuing the *glb-12* RNAi reduction in fecundity (*n*=5). Percentages show control RNAi compared to *glb-12* RNAi within a strain, *P<0.05; **P<0.01. Data are mean±s.e.m.

Because O_2_^.−^ is a very short-lived signalling molecule, we reasoned that GLB-12 would show a very specific tissue and subcellular localization to achieve an exact juxtaposition with potential downstream targets. Indeed, besides a very distinct expression pattern in the somatic gonad (Fig. 1c), the GLB-12 reporter was also found to be membrane-bound through a short N-terminal extension (Fig. 4a; Supplementary Fig. 4a). This extension harbours predicted sites for both myristoylation^23^ and palmitoylation^24^ (Fig. 4a), post-translational modifications that promote stable membrane attachment^25^. Deletion of these sites indeed prevented plasma membrane localization (Fig. 4b; Supplementary Fig. 4b). Myristoylation and palmitoylation are specifically associated with protein translocation to membrane rafts, which are dynamic membrane subdomains that compartmentalize cellular processes, including specific redox signalling events^26^. For GLB-12, we indeed observed a clustered distribution, both in the somatic gonad and in the nervous system (Supplementary Fig. 4c,d). Finally, a cytoplasmic version of the RNAi-resistant *glb-12* gene was not capable of rescuing the decreased fecundity following *glb-12* RNAi (Fig. 4c). Combined, these results support that the localization of GLB-12 serves as a spatial determinant for the O_2_^.−^ signal, both on a tissue and intracellular level.

### GLB-12 interacts with an intra- and extracellular SOD

**Figure 5:**
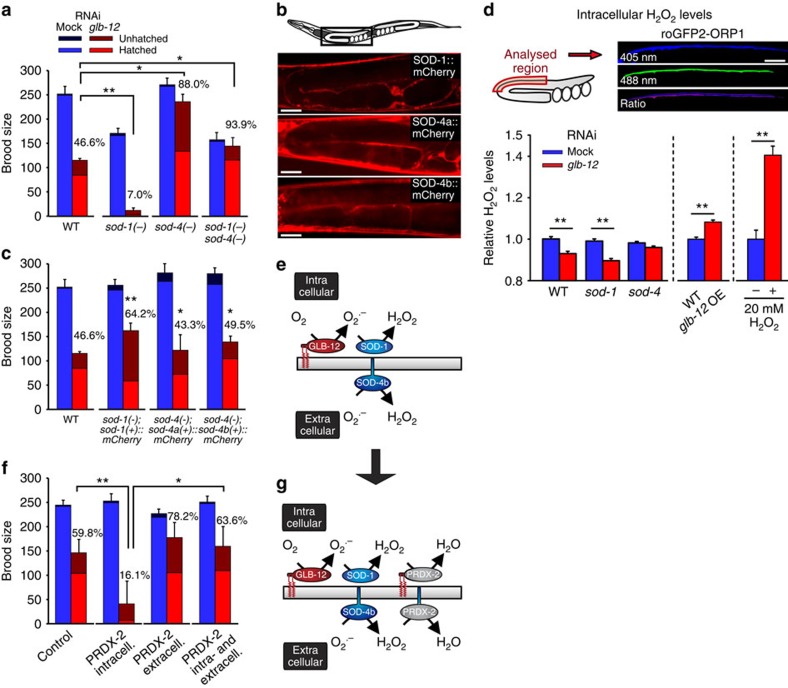
GLB-12 interacts with both an intra- and extracellular superoxide dismutase. (**a**) The effects of *glb-12* RNAi were aggravated in a mutant for the main cytoplasmic SOD-1 and reduced in a mutant for the extracellular SOD-4 (*n*=3). (**b**,**c**) SOD-1 and both isoforms of SOD-4 are present in the somatic gonad, and the SOD-1 and SOD-4 reporter constructs, when expressed in the *sod-1* and *sod-4* mutant, respectively, were able to revert the *glb-12* RNAi reduction in fecundity to WT levels (*n*=3). * in (**c**) shows significant differences for the strain carrying *sod-1::mCherry* compared with the *sod-1* strain and for the strains carrying *sod-4a/b::mCherry* compared to the *sod-4* strain. Scale bar: 20 μm. (**d**) Relative H_2_O_2_ levels in the somatic gonad were determined using the ratiometric probe roGFP2-ORP1. Depletion and overexpression of GLB-12 causes a decrease and increase, respectively, in intracellular H_2_O_2_ levels (*n*=6). Exposing worms to 20 mM H_2_O_2_ was included as a positive control (*n*=3). Scale bar: 10 μm. (**e**) Graphic showing the working model for GLB-12 signalling. (**f**) Introducing an intracellular and extracellular PRDX-2 mimics the effects seen for *glb-12* RNAi in mutants for SOD-1 and SOD-4, respectively (*n*=4). (**g**) The anticipated effect of artificially introducing the H_2_O_2_ scavenger PRDX-2 on GLB-12 signalling. *P<0.05; **P<0.01 (two-sided Student *t*-test). Data are represented as mean±s.e.m.

*In vivo*, O_2_^.−^ produced by GLB-12 may be converted into the more stable H_2_O_2_ by superoxide dismutases (SODs), H_2_O_2_ being an accepted messenger in redox signalling^1^,^27^. To test this hypothesis, we reduced GLB-12 levels in mutants for the five *C. elegans sod* genes (*sod-1* to *sod-5*) and found an aggravated effect on fecundity in the main cytoplasmic *sod-1* mutant and, surprisingly, a reduced effect in the extracellular *sod-4* mutant (Fig. 5a). Also in the second cytoplasmic *sod-5* mutant and, to a lesser extent, in the mitochondrial *sod-3* mutant an aggravated effect was observed (Supplementary Fig. 5a). Further, the effect in a *sod-1;sod-4* and a *sod-4* mutant are comparable, indicating that SOD-4 is epistatic to SOD-1 (Fig. 5a). Expression analysis of SOD-1 and both isoforms of SOD-4 showed that they are present in the entire somatic gonad (Fig. 5b, Supplementary Fig. 5b), thus overlapping in expression with GLB-12. When these reporter constructs were expressed in the corresponding *sod* mutant, the *glb-12* RNAi effect on worm fecundity largely reverted to levels observed in the WT (Fig. 5c). Targeting a H_2_O_2_-specific GFP-probe, roGFP2-ORP1 (ref. 28), to the intracellular location of GLB-12 showed that GLB-12 depletion indeed lowered H_2_O_2_ levels *in vivo* in the somatic gonad, while overexpressing GLB-12 increased H_2_O_2_ levels (Fig. 5d, Supplementary Fig. 5c). Although not statistically significant, H_2_O_2_ levels appeared to be further reduced in a *sod-1* background following GLB-12 depletion, again indicating that the intracellular SOD-1 converts GLB-12-produced O_2_^.−^ to H_2_O_2_ (Fig. 5d,e). Surprisingly, intracellular H_2_O_2_ levels in a *sod-4* background appeared to be less reduced compared to the WT following GLB-12 depletion (Fig. 5d), although this difference is again not statistically significant. The type of interaction between GLB-12 and SOD-4 is at this moment not clear; GLB-12-produced O_2_^.−^ could potentially penetrate the membrane through anion channels^29^ and be converted by the extracellular SOD-4, or O_2_^.−^ could be generated extracellularly by an unknown source. To further test the hypothesis that GLB-12/SOD-1 and SOD-4 work in parallel on both sides of the gonadal sheath cell membrane, we artificially targeted PRDX-2, an H_2_O_2_ scavenger, to both sides of the membrane (Fig. 5f,g; Supplementary Fig. 5d,e); following *glb-12* RNAi, the presence of PRDX-2 should then mimic the loss of SOD. This was indeed observed, with the strongest effect by the intracellular PRDX-2 (Fig. 5f). Taken together, these results strongly suggest that SOD-1 and SOD-4 modulate the downstream effects of GLB-12 in opposite ways. Endogenous occurring H_2_O_2_ scavengers like peroxiredoxins and catalases could further influence the GLB-12-based redox signal. However, loss of relevant peroxiredoxins and catalases did not affect the *glb-12* RNAi phenotype, indicating that these H_2_O_2_ scavengers do not interact with GLB-12 (Supplementary Fig. 5f). Surprisingly however, when both SOD-1 and each of these H_2_O_2_ scavengers are lost, brood sizes following *glb-12* RNAi are comparable to the WT and not to the SOD-1 single mutant (Supplementary Fig. 5f). This may suggest that lack of H_2_O_2_ scavenging becomes important when the GLB-12 redox signal is severely diminished, that is, when GLB-12 levels are reduced and SOD-1 is absent, but further analysis is needed to test this hypothesis. Finally, GLB-12 overexpression in the WT as well as in the *sod-1* or *sod-4* background did not result in an obvious effect on fecundity (Supplementary Fig. 5g), even though GLB-12 overexpression led to an increase in peroxide levels (Fig. 5d). Given the complexity of GLB-12-based signalling, negative feedback regulation might potentially be an explanation for this absence of a phenotype.

### GLB-12 modulates apoptosis via the p38/JNK MAPK pathways

**Figure 6:**
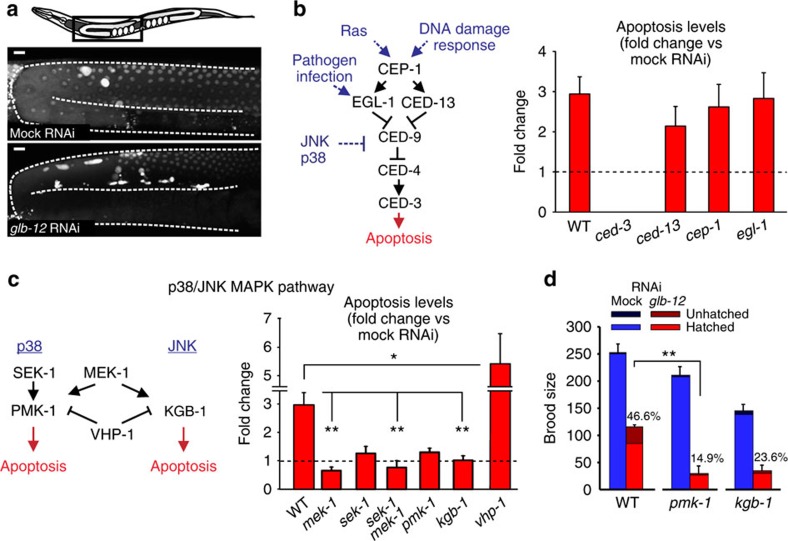
GLB-12 regulates apoptosis via the p38/JNK MAPK pathways. (**a**) Acridine orange staining was used to visualize the increase in germline apoptosis following *glb-12* RNAi. Scale bar: 10 μm. (**b**,**c**) *glb-12* RNAi caused an increase in apoptosis in the WT and in mutants for CEP-1, EGL-1 and CED-13, but not in mutants for the p38/JNK MAPK pathways (*n*=5). Bars show average apoptotic corpses per animal in *glb-12* RNAi treated animals, relative to control RNAi. The scheme shows the pathway associated with apoptosis (black) and upstream signalling pathways (blue), as well as a simplified version of the p38/JNK MAPK pathway. (**d**) *glb-12* RNAi caused a further decrease in fecundity in mutants for the p38/JNK MAPK pathways (*n*=3). *P<0.05; **P<0.01 (two-sided Student *t*-test). Data are represented as mean±s.e.m.

To identify potential downstream targets of GLB-12, we focused on its role in germline apoptosis, which is regulated by a well-characterized signalling cascade^30^. In WT worms, *glb-12* RNAi led to a doubling in germ cell corpses, while germline proliferation was not significantly affected under these conditions (Fig. 6a,b; Supplementary Table 1). In a *ced-3* mutant, in which germline apoptosis is absent, no corpses were present following both control and *glb-12* RNAi (Fig. 6b). An increase in germ cell corpses can be caused by an increase in germline apoptosis, or by a decrease in corpse engulfment and removal by the somatic gonad. Because *glb-12* RNAi still caused increased germline apoptosis in engulfment-defective mutants, did not affect the speed of corpse removal by the somatic gonad and had no visible effects on somatic gonad structure (Supplementary Fig. 6a–c), we concluded that GLB-12 is directly involved in germline apoptosis. In loss-of-function mutants for the pro-apoptotic genes *cep-1*, *ced-13* and *egl-1*, GLB-12 depletion still caused an increase in germline apoptosis (Fig. 6b; Supplementary Fig. 6d), indicating that GLB-12 does not signal through these proteins. The p38/JNK MAPK pathways act independently of CEP-1, CED-13 and EGL-1 (Fig. 6b) and are responsible for stress-induced germ cell apoptosis^31^. *glb-12* RNAi in loss-of-function mutants for these pathways no longer led to an increase in germ cell apoptosis (Fig. 6c; Supplementary Fig. 6e), while in a *vhp-1* mutant, an inhibiting phosphatase for these pathways^32^, *glb-12* RNAi caused a larger increase in germ cell apoptosis. Further, *glb-12* RNAi in the WT also increased the relative amount of phosphorylated PMK-1 (Supplementary Fig. 6f). Together, these results strongly suggest that GLB-12 has antiapoptotic effects by inhibiting the p38/JNK MAPK pathways. Surprisingly however, in *ced-3* or p38/JNK MAPK pathways mutants, fecundity was even further reduced following *glb-12* RNAi compared with WT and several gonadal defects could still be observed (Fig. 6d; Supplementary Fig. 6g,h). It therefore appears that the p38/JNK MAPK pathways do not mediate all downstream effects of GLB-12 on reproduction, suggesting that GLB-12 influences additional pathways.

**Figure 7:**
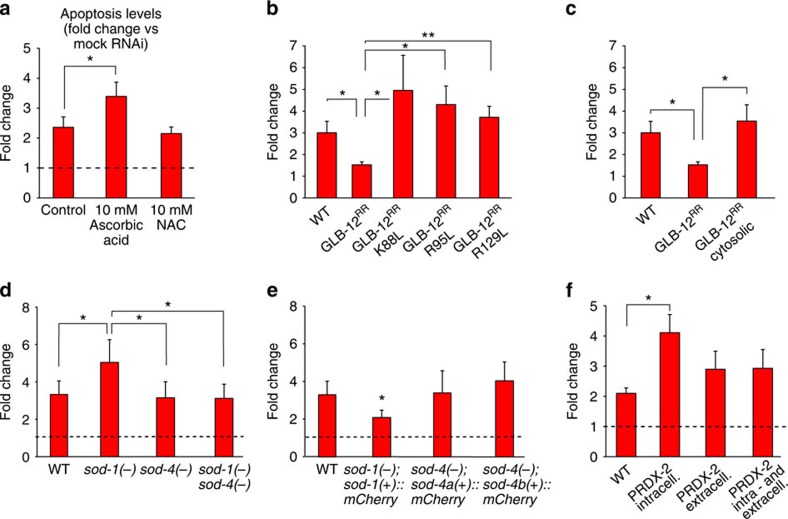
GLB-12 regulates apoptosis through redox signalling. (**a**) Ascorbic acid enhanced the *glb-12* RNAi increase in apoptosis (*n*=6). (**b**,**c**) The GLB-12 mutants incapable of O_2_^.−^ production and the cytosolic GLB-12 cannot rescue the apoptosis increase following *glb-12* RNAi (*n*=5). (**d**,**e**) SOD-1 deletion and overexpression enhance and decrease the apoptosis increase following *glb-12* RNAi, respectively (*n*=6). * in (**d**) indicates significant difference compared to the *sod-1* mutant. (**f**) Introducing an intracellular PRDX-2 appears to mimic the effects seen for *glb-12* RNAi in the mutant for SOD-1 (*n*=5). *P<0.05; **P<0.01 (two-sided Student *t*-test). Data are mean±s.e.m.

Finally, we assessed if GLB-12 influences germline apoptosis by its O_2_^.−^ redox signal. We indeed observed that one of the two antioxidants used, ascorbic acid, further augmented apoptosis levels following *glb-12* RNAi (Fig. 7a; Supplementary Fig. 7a), while the GLB-12 mutants incapable of producing O_2_^.−^ or without native GLB-12 localization were unable to rescue the increase in apoptosis following *glb-12* RNAi (Fig. 7b,c; Supplementary Fig. 7b,c). The antiapoptotic effect of GLB-12 also appears to be modulated by SOD-1 and SOD-4: loss of SOD-1, leading to a reduced conversion of O_2_^.−^ to H_2_O_2_, enhanced relative germline apoptosis levels following *glb-12* RNAi (Fig. 7d; Supplementary Fig. 7d), SOD-1 reporter expression in the *sod-1* mutant, which restores the conversion of O_2_^.−^ to H_2_O_2_, significantly suppressed the increase in germline apoptosis compared with *sod-1* worms following *glb-12* RNAi (Fig. 7e; Supplementary Fig. 7e) and artificially targeting the H_2_O_2_ scavenger PRDX-2 to colocalize with GLB-12, thereby again removing H_2_O_2_ and thus mimicking loss of SOD-1, further augmented apoptosis levels following *glb-12* RNAi (Fig. 7f; Supplementary Fig. 7f). Following *glb-12* RNAi, loss of the extracellular SOD-4 did not significantly affect apoptosis levels compared with WT worms. However, when both SOD-1 and SOD-4 were lost, apoptosis levels were lower compared to when only SOD-1 was lost and comparable to what is observed in WT animals (Fig. 7d; Supplementary Fig. 7d). This supports that both the intra- and extracellular H_2_O_2_ levels are important for GLB-12-based signalling. In line with this, scavenging H_2_O_2_ by targeting PRDX-2 to both the intra- and extracellular side appeared to lead to a lower increase in apoptosis following *glb-12* RNAi compared to when PRDX-2 was only present intracellular (Fig. 7f; Supplementary Fig. 7f). SOD-1 and SOD-4 loss also had opposite effects on the relative amount of phosphorylated PMK-1 following GLB-12 depletion, which further supports their opposite roles in GLB-12-mediated apoptosis (Supplementary Fig. 6f). In general, the role of SOD-1 and especially SOD-4 in GLB-12-mediated apoptosis appeared less pronounced compared to their role in GLB-12-mediated fecundity. This could indicate that other redundant factors are also involved in GLB-12-mediated apoptosis. Lastly, no added effect following *glb-12* RNAi was observed when natural occurring H_2_O_2_ scavengers catalase or peroxiredoxin were lost (Supplementary Fig. 7g). Surprisingly again, when each of these scavengers together with SOD-1 is lost, whereby not only less H_2_O_2_ is produced but also less is removed, the apoptosis increase is less severe than when only SOD-1 is lost (Supplementary Fig. 7h). These results are thus comparable to the effects of these double mutations on the brood size following GLB-12 depletion. As mentioned, the potential role of these H_2_O_2_ scavengers in GLB-12-based signalling is not clear at this moment.

## Discussion

**Figure 8:**
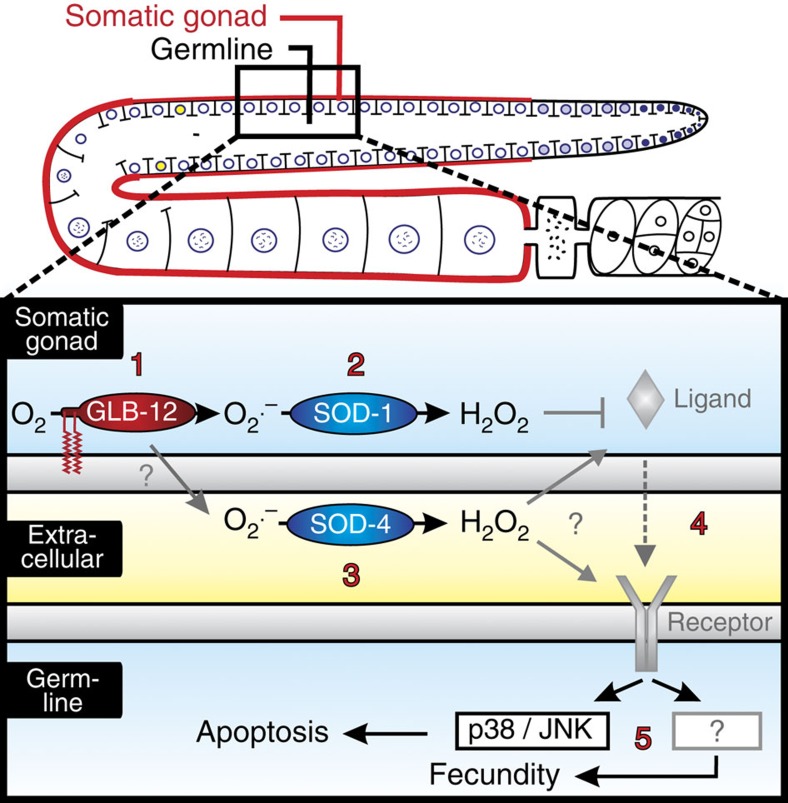
GLB-12 working model. GLB-12 is expressed in the somatic gonad and is capable of converting O_2_ to O_2_^.−^ (1) after which this signal is modulated by an intracellular SOD (2) and influenced by an extracellular SOD (3). Combined, GLB-12, SOD-1 and SOD-4 influence germline functioning, including p38 and JNK MAPK-mediated apoptosis (4–5).

In this study, we identified a novel role within the globin superfamily by showing that GLB-12 of *C. elegans* functions as a redox signalling protein in the reproductive system. Based on our findings, we propose a model whereby GLB-12 acts as a superoxide generator in the somatic gonad, after which this O_2_^.−^ signal is modulated directly or indirectly by an intracellular and an extracellular SOD, creating a transmembrane H_2_O_2_ gradient that acts as a redox signal. This signal then modulates reproduction, including p38/JNK MAPK-dependent germ cell apoptosis (Fig. 8).

The role of GLB-12 in redox chemistry is supported by its biochemical characteristics. We observed that GLB-12 shows a hexacoordinated haem, extremely low ligand affinity, a relatively low redox potential and the ability to transfer electrons to other molecules in a continuous manner. Haem hexacoordination in globins has previously been associated with increased redox kinetics for the haem iron^9^,^10^,^11^, and, as a result, hexacoordinated globins have been hypothesized to function in redox biology^7^. For example, hexacoordinated globins have been proposed to function in electron transfer^33^,^34^, to work as redox sensors to control behaviour^9^, to regulate lipid oxidation^35^ or to function as NO scavengers^36^,^37^. However, it has thus far been very difficult to link these observations to the biochemical mechanisms used by these globins *in vivo*.

We found that, by using structural, biochemical and mutational approaches, GLB-12 actively converts O_2_ to O_2_^.−^, which in turn is used as a redox signalling molecule. As this hexacoordinated globin can actively support redox signalling, it provides a reference model for the function of globins and globin-like proteins in other organisms.

It is already well appreciated that redox signals can originate from endogenous sources, such as membrane-bound NADPH oxidases or mitochondria, but the control mechanisms on ROS as endogenous signalling molecules are still poorly understood. ROS signal specificity can be achieved by strict compartmentalization: by tightly controlled spatio-temporal expression of the ROS generators in proximity to the downstream target, efficient signal transduction can be realized^4^. This specificity in spatial expression is indeed observed for GLB-12: (1) at the tissue level, GLB-12 is only present in a specific part of the somatic gonad, wherefrom it acts to regulate germline reproduction; (2) at the subcellular level, GLB-12 is bound to the plasma membrane by myristoylation and palmitoylation, whereby this localization increases the effectiveness of the GLB-12 signal. In addition, these types of protein acylation localize proteins to dynamic membrane subdomains that have been associated with specific redox signalling events^26^. Given that GLB-12 produces O_2_^.−^ and that O_2_^.−^ is diffusible and short lived, we therefore reason that the location of GLB-12 serves as a spatial determinant for downstream signals, both on a tissue and intracellular level. In this context, our observation that GLB-12 shows a 40-fold lower O_2_^.−^ production rate compared with cytochrome P450 might indicate that high ROS levels are not required when the redox signal is strictly localized.

On top of localizing the enzymatic source of a redox signal with its potential downstream target, an additional layer of regulation can be achieved by alterations in the local reduction/oxidation capacity^4^. We observed that GLB-12 acts together with the main cytoplasmic SOD-1, whereby the latter enhances the spontaneous oxidation of O_2_^.−^ into O_2_ and H_2_O_2_, and this interaction indeed increases the effectiveness of the H_2_O_2_ redox signal. To our surprise, also the extracellular SOD-4 appears to influence the GLB-12 signal and this in an opposite manner compared to SOD-1. These results clearly show that SODs have essential roles in the regulation of redox signalling and are not merely generic antioxidants. We hypothesize that H_2_O_2_, produced by GLB-12/SOD-1, influences ligand release from the somatic gonad, while H_2_O_2_ produced by SOD-4 could either influence ligand release or affect receptor activity on the germline plasma membrane (Fig. 8). These observations are in line with the increasing amount of evidence that the intra- and extracellular redox states work in concert to influence cell signalling^38^,^39^. Furthermore, the interaction between GLB-12 and these two SODs adds a surprising third level of regulation in this redox signal, whereby the amount of O_2_^.−^ and H_2_O_2_ on the intra- and extracellular side of the plasma membrane are important determinants of the downstream signal. It is interesting to note that, while plasma membranes form a physical barrier to ROS, ROS transport across membranes could occur via selective membrane channels. This has been observed for both O_2_^.−^ and H_2_O_2_ with certain classes of anion channels^29^ and aquaporins^4^,^40^,^41^,^42^. This ROS transport across membranes has therefore been hypothesized to participate in the regulation of redox signalling and could also be involved in GLB-12-mediated signalling. Overall, our results show that redox signalling by GLB-12 is regulated by multiple control layers. Interesting to note is that NADPH oxidase, a known ROS generator, also displays a very specific subcellular localization that is related to its function, and also appears to interact with SOD enzymes^26^,^43^. These shared characteristics between GLB-12 and NADPH oxidases indicate that redox signalling proteins function according to similar principles. Furthermore, the presence of a transmembrane redox gradient in GLB-12-mediated signalling presents a fascinating additional principle in how redox signalling can be further modulated.

We linked the redox signalling function of GLB-12 with *C. elegans* reproduction. Reduction of GLB-12 levels causes decreased fecundity, smaller gonads, increased germline apoptosis levels and several defects during oocyte development. We further showed that GLB-12 has antiapoptotic effects by inhibiting the p38/JNK MAPK pathways. These highly conserved pathways have been shown to be sensitive to environmental stress, including oxidative stress, in several organisms^44^,^45^. Also in *C. elegans*, their combined role in modulating germ cell apoptosis in response to stressors such as oxidative and heavy metal toxicity has been demonstrated^31^. The results presented here provide a link between a redox signalling protein and p38/JNK MAPK-mediated germline apoptosis. Importantly, GLB-12-based redox regulation of the p38/JNK MAPK pathways, using low local levels of superoxide, is opposite from the effects of excessive oxidative stress on this pathway. Because GLB-12 acts specifically from the somatic gonad and not from the germline, it likely influences the release of ligands and/or the activation of receptors of the p38/JNK MAPK pathways. In other organisms, it has indeed been observed that ROS can influence receptor activation upstream of the p38/JNK MAPK pathways^44^. Our results presented here indicate that, when p38/JNK MAPK ligands and receptors in the *C. elegans* reproductive system will be identified, they could form an interesting model to study how GLB-12-mediated redox signalling can influence the activity of downstream signalling cascades. Finally, in *C. elegans*, about half of all germ cells die by p53-independent ‘physiological' apoptosis, and it is thought that this process increases the quality of the surviving germ cells by the relocation of cytoplasmic components^46^. As fecundity is further reduced by combining *glb-12* RNAi with p38/JNK pathway inhibition, GLB-12 may be required to generate or allocate this cytoplasmic material via modulation of germline apoptosis levels. In the absence of GLB-12, apoptosis is then increased as a compensatory measure to sustain reproduction, albeit at lower levels. This would explain why preventing the increase in cell death during GLB-12 depletion leads to a further decrease in brood size.

In conclusion, we have identified a globin that acts as a source of redox signalling. The specific biochemical characteristics of GLB-12 show how a globin can fulfil this function, while the precise subcellular localization and the interaction with the SOD antioxidant enzymes increase the effectiveness of the redox signal. The opposite effect of the intra- and extracellular SOD on GLB-12 signalling is particularly intriguing; it indicates the importance of transmembrane redox gradients and further deepens the concepts of redox signalling compartmentalization. The notion that highly localized ROS gradients influence important biological processes such as reproduction could open up a new area of redox biology. Finally, given the widespread occurrence of globins, the majority with unknown function, our results add an important member to the group of proteins that drive redox signalling.

## Methods

### General methods and strains

Maintenance of *C. elegans* was carried out according to standard procedures. *C. elegans* strains were cultured at 20 °C on cholesterol-supplemented nutrient agar (OXOID) plates containing a lawn of freshly grown *Escherichia coli* K12 cells. To obtain synchronized cultures, gravid adults were lysed by hypochlorite treatment and eggs were allowed to hatch overnight in S buffer. Worm strains used in this study were Bristol N2 wild type, NL2099 [*rrf-3(pk1426)* II], TU3311 [uIs60], NL3511 [*ppw-1(pk1425)* I], RB798 [*rrf-1(ok589)* I], OD95 [*unc-119(ed3)* III; ltIs37 IV; ltIs38], GA187 [*sod-1(tm776)* II], GA184 [*sod-2(tgk257)* II], GA186 [*sod-3(tm760)* X], GA416 [*sod-4(gk101)* III], GA502 [*sod-5(tm1146)* II], RB1653 [*ctl-3(ok2042)* II], VC1151 [*prdx-3(gk529)* III], VC289 [*prdx-2(gk169)* II], MT1522 [*ced-3(n717)* IV], FX536 [*ced-13(tm536)* X], TJ1 [*cep-1(gk138)* I], MT8735 [*egl-1(n1084n3082)* V], FK171 [*mek-1(ks54)* X], KU4 [*sek-1(km4)* X], *mek-1(ks54) sek-1(km4),* JT366 [*vhp-1(sa366)* II], KB3 [*kgb-1(um3)* IV], KU25 [*pmk-1(km25)* IV], HT1593 [*unc-119(ed3)* III], EG6699 [ttTi5605 II; *unc-119(ed3)* III; oxEx1578], which were provided by the *Caenorhabditis* Genetics Center funded by the National Institutes of Health National Center for Research Resources. Standard crossing was used to generate double mutants.

### Molecular biology

Translational reporters and genetic constructs were made using fusion PCR^47^, whereby 20 bp overlapping extensions were used to stitch PCR products together. Individual PCR products were first amplified separately, purified and used as template for the subsequent fusion PCR reaction at ∼0.1–1 ng per 50 μl PCR reaction. Final PCR products were also purified and injected into the gonads of young adult hermaphrodites (HT1593 [*unc-119(ed3)* III]) to generate extrachromosomal arrays, whereby a PCR product coding for the *unc-119* gene was used as a co-injection marker. Primer sequences can be found in Supplementary Table 2. Because we observed that the inherent expression variation that is associated with extrachromosomal arrays can be seen over different animals within each injected line and that different lines show the same type of variation, 3–5 hermaphrodites were injected for each construct and the resulting transgenic lines were pooled. Expression analysis was performed on at least 10 animals per construct and representative images are shown. For brood size and germline apoptosis analysis, respectively at least 6 or 10 animals were analysed per transgenic line, per condition and per biological replicate.

The translational reporters contain the target gene and ∼3 kb upstream and 0.5 kb downstream of the target gene, to include endogenous promoter and 3′UTR elements. For the different constructs, the exact size of these regions can be found in the Supplementary Figures, in the graphics describing these constructs. The *gfp* or *mCherry* gene was fused at the 3′ side of the target gene, and was preceded by the sequence 5′ggagctggtgcaggcgctggagccggtgcc 3′ coding for a GA-linker region. Translational reporter constructs were injected at a concentration of 50 ng μl^−1^, together with the *unc-119* gene at a concentration of 20 ng μl^−1^. Transformed reporter lines were analysed using a Nikon Eclipse TE2000-5 confocal microscope.

An RNAi-resistent *glb-12* gene (*glb-12*^*RR*^) was generated using the guidelines described by Green *et al.*^48^ In short, the nucleotide sequence was recoded by shuffling alternative codons encoding the same amino acid, without changing the original amino acid sequence or the overall codon bias, until there were no long (>9 bp) stretches of homology between the original and recoded *glb-12* gene. The five *glb-12* introns were replaced by the three synthetic introns that are also found in eGFP. The *glb-12*^*RR*^ gene was fused at the 3′ side to the coding sequence for the self-cleaving peptide T2A^49^, 5′gagggcagaggaagtctgctaacatgcggtgacgtcgaggagaatcctggccca 3′, followed by the *gfp* gene. When the ribosome encounters the T2A sequence, a ‘ribosomal-skip' or ‘STOP&GO' occurs, releasing the GLB-12 polypeptide while translation of the messenger RNA for GFP continues. This results in the simultaneous expression of GLB-12 and GFP, even though the two proteins are not fused together. The entire construct was fused to the same up- and downstream regions used for the *glb-12* translational reporter. The exact size of these different elements for this construct is shown schematically and can be found in the Supplementary Figures. The *glb-12*^*RR*^ genes coding for the mutants K88L, R95L and R129L were generated by site-directed mutagenesis. The cytosolic *glb-12*^*RR*^ gene was generated by removing the region coding for the first 30 AA of GLB-12, which is the N-terminal membrane-targeting region. All *glb-12*^*RR*^ genes were validated by sequencing.

The constructs coding for the intracellular and extracellular targeted PRDX-2 were generated by combining the *prdx-2* gene with the regulatory and coding regions of the *glb-12* gene and the *sod-4b* gene, respectively. These construct artificially target PRDX-2 to the intracellular location of GLB-12 or extracellular location of SOD-4B, respectively. These constructs are schematically described in the Supplementary Figures. These schemes also show the exact size of the different elements.

The gene coding for roGFP2-ORP1 was codon optimized with the web tool, *C. elegans* codon adapter^50^, to a codon adaptation index of 0.6 and with the introduction of three artificial introns. The resulting gene was fused to the upstream and downstream region of the *glb-12* gene, together with the sequence coding for the first 30 AA of GLB-12. This construct targets the roGFP2-ORP1 probe to the intracellular location of GLB-12. Also this construct is schematically represented in the Supplementary Figures.

We observed that the presence of the four different *glb-12* introns, in combination with the *glb-12* upstream and downstream region, were essential for somatic gonad expression. These introns were separately amplified as short PCR fragments with the following oligo's: 5′gtgagttttgagcttgattc 3′ and 5′ccgacttgctggaaaataat 3′ for intron 1, 5′gagttcatggagcaggttag 3′ and 5′tctggttctgattttgttcca 3′ for intron 2 and 3, and 5′tgagattgtgggatcagt 3′ and 5′aaatcatatttgttgggtga 3′ for intron 4. The resulting PCR products were co-injected with all constructs that carry the *glb-12* upstream and downstream region but lack these intronic regions: all *glb-12*^*RR*^ constructs, the intracellular *prdx-2* construct and the *roGFP2-orp1* construct. The *glb-12* introns were co-injected at 2–6 ng μl^−1^. The *glb-12*^*RR*^ and *prdx-2* constructs were injected at 25 ng μl^−1^, together with 20 ng μl^−1^ for the *unc-119* gene. A control strain was generated by only injecting 20 ng μl^−1^ for the *unc-119* gene. The *roGFP2-orp1* construct was injected at 50 ng per μl, together with 20 ng μl^−1^ for the *unc-119* gene. To create the transgenic strain overexpressing *glb-12* together with *roGFP2-orp1*, a PCR product of the *glb-12* gene, including the 2,120 bp upstream and 473 bp downstream region of the *glb-12* gene, was co-injected at 10 ng μl^−1^. The transgenic strain carrying only the *roGFP2-orp1* construct was further crossed in the strains GA187 [*sod-1(tm776)* II] and GA416 [*sod-4(gk101)* III]. To create the transgenic strain overexpressing *glb-12*, a PCR product of the *glb-12* gene, including the 2,120 bp upstream and 473 bp downstream region of the *glb-12* gene, was injected at 10 ng μl^−1^, together with 20 ng μl^−1^ for the *unc-119* gene in the *unc-119* mutant. For the control strain, we only injected the *unc-119* rescue gene in the *unc-119* mutant. To overexpress GLB-12 in a *sod-1* and *sod-4* mutant background, we injected the endogenous *glb-12* gene (10 ng μl^−1^) together with a co-injection marker, *Pmyo-2::mCherry::unc-54UTR* (2.5 ng μl^−1^), in both these mutants. The control strains were generated by only injecting the *Pmyo-2::mCherry* marker. To assess if GLB-12 is expressed in the germline, the GLB-12 translational reporter described above, including the 2,120 bp upstream and 473 bp downstream region of the *glb-12* gene was cloned in the pCFJ150 vector by Gibson cloning and injected into strain EG6699[ttTi5605 II; *unc-119(ed3)* III; oxEx1578] to create integrated single-copy transgenes using the MosSCI method^51^. This GLB-12 reporter thus carries the endogenous promoter, intronic and 3′UTR regions, which should mimic endogenous expression as closely as possible. Furthermore, the MosSCI integration site ttTi5605 in the strain EG6699 is known to allow stable germline expression of integrated constructs carrying regulatory regions that drive germline expression^51^. Besides a much weaker expression, the integrated reporter showed a comparable expression pattern compared with the extrachromosomal GLB-12 translational reporter. No expression was seen within the germline. For localization in human neuroblastoma SH-SY5Y cells, cDNAs of wt *glb-12*, *glb-12*Δmyr, *glb-12*Δpalm, and *glb-12*ΔmyrΔpalm were cloned into the pEGFP-N1 vector (Clontech) using BglII and HindIII restriction enzymes (Biolabs, Westburg). Ligation of cDNAs in the vectors was performed using T4 DNA ligase (Novagen). The myristoylation site, glycine at position 2, was modified to alanine and is annotated as GLB-12Δmyr. The palmitoylation site, cysteine at position 6, was mutated to alanine and is annotated as GLB-12Δpalm. The mutant where both fatty acylation sites are mutated to alanine is annotated as GLB-12ΔmyrΔpalm. These same mutations were also introduced in the GLB-12 translational reporter for *C. elegans*, which has been described above. All mutations were done with the Quickchange site directed mutagenesis kit (Stratagene) and validated by sequencing.

Human neuroblastoma SH-SY5Y cells (ATCC CRL-2266) were cultured as recommended by the manufacturer's protocol. Cells were transfected with 3 μl of lipofectamine 2,000 and 0.5 μg of pEGFP-N1 plasmide. After 4 h, transfection medium was replaced by growth medium and SH-SY5Y-cells were allowed to express the GFP-tagged proteins for 24 h. For GLB-12Δpalm, colocalization was performed with the MitoTracker Deep Red probe (Invitrogen) according to the manufacturer's protocol. Localization was examined with an UltraVIEW Vox ERS microscope (Perkin-Elmer), and images were created with the Volocity 6.0.1 software.

### Feeding RNAi

RNAi was applied by feeding bacteria expressing dsRNA to the worms. The RNAi clone targeting *glb-12* was derived from the Ahringer RNAi library^13^. An *in silico* analysis of the specificity of *glb-12* RNAi with the online tool E-RNAi^52^ presented no aspecific targets. RNAi induction was performed as described by Timmons *et al.*^53^: an overnight culture of RNAi bacteria was inoculated in LB medium with 100 μg ml^−1^ ampicilline and 12.5 μg ml^−1^ tetracycline and incubated at 37 °C with shaking. The following day, cultures were diluted 1:100 and grown to OD595=0.4 at 37 °C with shaking, after which sterile IPTG was added to a final concentration of 0.4 mM. Cultures were again incubated at 37 °C with shaking for 4 h, and finally spiked with another 100 μg ml^−1^ ampicilline and 12.5 μg ml^−1^ tetracycline. IPTG was added to a final concentration of 0.8 mM. NGM plates with 100 μg ml^−1^ ampicilline, 12.5 μg ml^−1^ tetracycline and 0.4 mM IPTG were seeded with the bacterial cultures, air dried and left overnight at room temperature. Finally, synchronized L1 worms were placed on these NGM plates seeded with the freshly induced RNAi bacteria.

### Fecundity assay

Two days after synchronized L1 worms were placed on plates containing RNAi bacteria, individual L4 hermaphrodite were transferred to NGM plates with a 25 μl spot of four times concentrated, induced RNAi bacteria and maintained at 20 °C, except for the temperature-sensitive strain *rrf-3 (pk1426)*, which was kept at 17 °C. Animals were transferred to fresh plates each day until they stopped laying eggs. Twenty-four hours after adults were transferred, hatched larvae and unhatched eggs on each plate were counted. The fecundity of each worm was calculated as the total number of hatched and unhatched eggs produced. At least 18 worms evenly spread over at least three independent replicas were analysed for each strain and for each condition. Afterwards, fecundity percentages of *glb-12* RNAi compared with control RNAi were calculated for each biological replicate within each genotype, after which statistical analysis was performed on fecundity percentages between genotypes.

### Germline apoptosis assay

For all germline apoptosis assays, one-day-old adult animals were used. Animals with clearly underdeveloped or abnormal looking gonads were not included in this analysis. Apoptotic cells observed in the late pachytene region of the germline were scored. For all germline apoptotis assays, at least 10 worms per biological replicate were scored, one gonadal arm per animal was used, and at least three biological replicates were carried out for each condition.

Apoptotic germ cells were visualized by acridin orange (AO) staining, unless otherwise noted. AO (1 mg ml^−1^) was added to a 9-cm diameter NGM plate carrying first-day adult animals for overnight incubation. The following day, worms were rinsed from the plate, washed, mounted under coverslips in 20 μl of a 12.5-mM sodium azide solution, and immediately analysed. The strain MT1522 [*ced-3(n717)* IV] was used to validate the specific staining of apoptotic cells by AO. Differential interference contrast microscopy instead of acridin orange staining was used to identify apoptotic germ cells in engulfment-defective mutants, as well as in WT animals that were used to analyse the removal speed of apoptotic corpses. Finally, for the latter experiment, animals were anaesthetized for 10 min with 10 mM levamisole instead of sodium azide. After this, animals were mounted on agar pads for analysis and time-lapse contrast microscopy was performed with through-focus z-stack images every 4 min, for a total of 75 min.

### Ascorbic acid and N-acetylcysteine treatment

Ascorbic acid and N-acetylcysteine were added into NGM media from a freshly made 100 mM stock solution the day before the plates were used. Worms were placed on plates containing these antioxidants starting from the fourth larval stage on.

### *In vitro* superoxide measurements

To test the capacity of haem proteins to produce superoxide *in vitro*, we applied the method of Hayashi *et al.*^16^ to create a reducing environment for haem proteins, and included lucigenin that produces light upon reaction with superoxide, to quantify superoxide production. In brief, the components of the reduction system are an NADPH-generating system, consisting of glucose-6-phosphate, glucose-6-phosphate dehydrogenase and NADP^+^ and an electron-mediating system, consisting of ferredoxin and ferredoxin-NADP reductase. We omitted catalase from the original system, as this might lead to quick removal of the produced superoxide. Concentrations of the different components, including the different haem proteins tested here, were as described in the original paper. haem proteins tested were myoglobin from horse heart (Sigma M1882), cytochrome P450 2B4 from rabbit liver (Sigma C7552) and cytochrome C from horse heart (Sigma C2506). Lucigenin was added to a final concentration of 100 μM. Measurements were performed in a 200-μl working volume in a 96-well microtiter plate. Glucose-6-phosphate was added last to start the reduction. Immediately following this, light emission was recorded in a Victor2 1420 Multilabel Counter (Perkin-Elmer, Wellesley, MA), whereby for each condition the average of 20 consecutive measurements was taken. Runs were repeated three times to allow statistical analysis.

### *In vivo* hydrogen peroxide measurements

A GFP-based probe specific for hydrogen peroxide, roGFP2-ORP1 (ref. 28), was used to measure relative levels of *in vivo* hydrogen peroxide levels at the subcellular location of GLB-12 and this specifically within the somatic gonad. To this end, a transgenic *C. elegans* line was generated that expressed roGFP2-ORP1 under the control of the *glb-12* regulatory regions, as described above.

To increase roGFP2-ORP1 expression levels, animals were cultivated at 22 °C instead of 20 °C. Hydrogen peroxide levels were measured in first-day adult hermaphrodites. Animals were removed from the plate, anaesthetised in a fresh mixture of 10 mM levamisol dissolved in M9 for 15–30 min before transferring them to an agarose pad under a coverslip for imaging. To measure *in vivo* hydrogen peroxide levels following exposure to 20 mM hydrogen peroxide, a freshly made hydrogen peroxide solution was added to the animals already placed on the agarose pad. Imaging started immediately following this and was completed within 10 min.

Animals were imaged using an inverted Zeiss LSM510 confocal microscope equipped with × 40 magnification objective lens (oil immersion) and Zeiss Zen 2009 software. Laser intensities and microscope settings that were compatible with ratiometric measurements were determined in an initial run and were kept constant for all subsequent experiments. Excitation of roGFP2-ORP1 was performed sequentially by 405 and 488 nm laser lines, emission was detected at 500–550 nm. Only those animals that showed clearly visible expression of roGFP2-ORP1 in the somatic gonad were used for image analysis. One or two single focal plane images were taken for each animal. At least five worms per biological replicate and per condition were scored, one gonadal arm per animal was used, and at least 3 biological replicates were carried out for each condition.

Image processing was based on the method used by Morgan *et al.*^54^ In brief, images were saved as 16-bit tiff files and further processed by ImageJ. Images were first converted to 32-bit tiff files, the background of both 405 nm images and 488 nm images was subtracted using an upper and lower threshold and background values were set to ‘not a number' (NaN). Relative H_2_O_2_ levels in these processed images were quantified by (1) selecting a polygonal region, which showed the probe within the somatic gonad and which showed no interfering background fluorescence from the gut, (2) calculating the average pixel intensities within this region for both the 405 nm and the 488 nm image and (3) calculating the ratio of the 405 nm average pixel intensity over the 488 nm average pixel intensity.

### Recombinant expression of GLB-12

To express recombinant GLB-12, young adult worms were collected and total RNA was prepared using the TriZol method (Invitrogen) followed by LiCl precipitation (Ambion). cDNA was prepared using the OneStep RT-PCR kit (Qiagen). Cycling conditions were as followed: 30 min at 50 °C for the RT reaction, followed by 15 min at 95 °C for the activation of the HotStar Taq DNA polymerase, followed by 35 cycles of 60 sec at 94 °C, 60 s at 54 °C and 90 s at 72 °C. cDNA of *glb-12* was cloned into the pET23a-vector (Novagen) using NdeI and XhoI restriction enzymes (Biolabs) and T4 Ligase (Novagen). The expression plasmid was then transformed into *Escherichia coli* strain BL21(DE3)pLysS (Invitrogen). Cells were grown at 25 °C in TB medium containing 200 μg ml^−1^ ampicillin, 30 μg ml^−1^ chloramphenicol and 1 mM δ-amino-levulinic acid. The culture was induced at A_550_=0.8 OD with IPTG (final concentration 0.04 mM). After overnight growth, *E. coli* cells were collected. Recombinant GLB-12 was spectroscopically localized in the cytosolic fraction and was purified to homogeneity using (i) ammonium sulfate precipitation, after which the 90% pellet was dissolved and dialyzed against 5 mM Tris-HCl pH 8.5, (ii) DEAE-Sepharose fast flow chromatography and (iii) Sephacryl S200 gel filtration in 50 mM Tris-HCl pH 8.5, 150 mM NaCl, 0.5 mM EDTA. The fractions containing the pure globin were pooled and concentrated.

### UV–vis

UV–vis spectra were measured in a 250–700 nm range on a Cary-5 UV–vis–NIR spectrophotometer (Varian). Ferrous CO-bound and reduced deoxy protein samples were prepared by flushing 1 ml of 100 mM potassium phosphate buffer pH 7.0 for 15 min with CO- and N_2_-gas, respectively, in a sealed cuvette. After addition of 10 μl of a satured solution of sodium dithionite, highly concentrated, recombinant purified protein was added to the flushed sealed cuvette to obtain a final concentration of 50 μM. Ferric CN-ligated GLB-12 was prepared by adding an excess of 40 mM of KCN.

### Cyclic voltammetry

Cyclic Voltammetry was performed using electrodes modified with a gelatin layer. The presence of this gelatin layer allows the incorporation of proteins and so the analysis of their electrochemical behaviour. Measurements were performed in a three electrode cell using a SCE containing two compartments (Radiometer Analytical, France) and a platinum counter electrode. The working electrodes were gold electrodes with a diameter of 1.6 mm (BAS, UK) which were pretreated by mechanical and electrochemical polishing according to the following procedure. Before its first use the electrode surface was briefly scoured by a silicon carbide emery paper of 1,200 grit to obtain a fresh surface. To smoothen the resulting relatively rough surface it was further subjected to sequential polishing by polishing cloth covered with alumina powder of 1, 0.3 and 0.05 mm particle size (Buehler, USA) for, respectively, 5, 10 and 20 min. To remove any adherent Al_2_O_3_ particles the electrode surface was rinsed thoroughly with doubly deionised water and cleaned in an ultrasonic bath containing deionised water (Branson 3210, USA) for 2 min. Before immobilizing gelatin onto the electrode, the gold surface was modified with a self-assembled monolayer (SAM) of 6-mercaptohexanol (unless otherwise indicated). The latter was done by immersing the electrode in a water solution containing 1 mmol L^−1^ 6-mercaptohexanol (MH) for 18 h at room temperature. The modified gold electrodes were consequently rinsed with water to remove any physically adsorbed mercaptohexanol.

To immobilize gelatin onto the electrode, gelatin B (gelB) powder (5 m%) was dissolved in 10 mM HEPES buffer solution at 40 °C and further mixed with globin (GLB) and/or cytochrome C peroxidase (CCP) solutions (50 μM). An amount of 7 μl of this gelatin solution was brought onto the surface of the electrode by using a syringe. The electrode was then exposed to air for 2 h at 4 °C (drop drying). The final electrodes are denoted as GLB|CCP|GelB (1.5:1.5:7 ratio), GLB|GelB (3:7 ratio) or CCP|GelB (3:7 ratio). Additional optimization of this analysis showed that highly comparable results can be obtained when proteins are dissolved in a small sample volume instead of a gelatin layer. Cyclic voltammetry for dissolved protein samples was conducted in an electrochemical cell designed by Hagen^55^. A droplet of the protein solutions (10–50 μM) was placed in the cell equipped with a Pt-counter and a SCE electrodes. Gold disk electrodes (diameter 1.6 mm, BASi, USA) modified with 6-mercaptohexanol (Sigma-Aldrich) were used as working electrodes.

A gelatin layer was used to determine the redox potential of GLB-12, the effect of varying pH on this, and the interaction of GLB-12 with CCP. Except for the pH depence, these measurements were repeated using dissolved protein samples and showed highly comparable results. All other measurements were performed on dissolved protein samples. All measurements were carried out under nitrogen atmosphere at room temperature (22±2 °C).

### Real-time quantitative RT-PCR

Total RNA was isolated from harvested *C. elegans* worms using the RNeasy Midi Kit from Qiagen (Hilden, Germany), according to the manufacturer's instructions. A DNase I (Zymo Research, Orange, California) digestion step was subsequently performed to remove genomic DNA. First strand cDNA was synthesized using an oligo(dT) primer and a Moloney murine leukemia virus reverse transcriptase (Fermentas, Vilnius, Lithuania).

Quantitative RT-PCR was carried out using a Rotor-Gene 2000 centrifugal real-time cycler (Corbett Research, Mortlake, Australia) using the Platinum SYBR Green qPCR SuperMix-UDG (Invitrogen). Each reaction contained: 12.5 μl of the Platinum SYBR Green qPCR SuperMix-UDG, 200 nM of forward and reverse primers and 5 μl cDNA (1:40 RNA dilution), to a final volume of 25 μl. Amplification was performed in 0.1 ml real-time PCR tubes (Corbett Research) placed in the 72-well rotor of the Rotor-Gene instrument. The cycling conditions were as follows: 50 °C for 2 min, initial denaturation at 95 °C for 2 min, followed by 45 cycles of 15 s at 95 °C, 30 s at 60 °C and 30 s at 72 °C (gain set at 8 for SYBR Green). Following the final cycle, melting curve analysis was performed to examine the PCR specificity in each reaction tube (absence of primer dimers and other nonspecific products). All PCR reactions were performed in triplicate. *Ct* values were transformed to relative quantities and were normalized using the geometric mean of three reference genes^56^. Four biological replicates were carried out for each experiment. Primer sequences can be found in Supplementary Table 2.

### Crystallization and structure determination

Atomic coordinates and structure factors determined from the GLB-12 crystal have been deposited in the Protein Data Bank, with entry code 4bja. Crystals of GLB-12 were grown at 4 °C by the hanging-drop vapour diffusion method, by mixing 1 μl protein solution with 1 μl crystallization well solution containing 2 M (NH_4_)_2_SO_4_, 0.2 M Na-acetate, 0.1 M Na-acetate pH 4.6. Crystals usually grew in about two months, and were cryo-protected with the same crystallization well solution supplemented with 30% glycerol, prior to cryo-cooling in liquid nitrogen. The crystals belong to the space group *P*6_5_22, with unit cell parameters *a*=50.4 Å, *b*=50.4 Å, *c*=245.3 Å, γ=120°, and one GLB-12 molecule per asymmetric unit. Diffraction data were collected to 1.65 Å resolution using synchrotron radiation (BM14 beamline, ESRF, Grenoble, France). Raw data were processed with Mosflm^57^ and Scala^58^ (Table 1).

The structure was solved by single-wavelength anomalous dispersion (SAD) method, based on the haem-Fe atom anomalous signal. The haem-Fe atom position and the initial phases were calculated by using the AutoSol pipeline implemented in PHENIX^59^. The GLB-12 model was built with the program COOT^60^ and restrained refined to the maximum resolution using REFMAC^61^. At the end of the refinement stages (including anisotropic B-factor refinement), 110 water molecules, 1 sulfate ion, and 4 acetate molecules were located through inspection of difference Fourier maps. The final Rfactor value was 17.8%, and Rfree 23.4%. No electron density was detected for residues 1–18, 167–181 and 216–266. The programs Procheck^62^ and Surfnet^63^ were used to assess the stereochemical quality of the protein structures and to explore the protein matrix cavities.

### TEM—high-pressure freezing and freeze-substitution

To analyse the subcellular localization of GLB-12, the GLB-12 translational reporter in combination with anti-GFP antibodies was used. N2 WT worms were included as negative control.

Copper membrane carriers (1.5 mm × 0.2 mm) (Leica, Microsystems, Vienna, Austria) treated with 1% lecithin were used and filled with 20% (w/v) Bovine Serum Albumin (BSA). Three to four one-day-old adult nematodes were transferred to the membrane carrier, immersed in the BSA and then immediately frozen in a high-pressure freezer (EM PACT, Leica, Microsystems).

Freeze substitution was carried out using a Leica EM AFS (Leica, Microsystems). Carriers were transferred from EM pact to AFS under liquid nitrogen and placed in an eppendorf filled with dry acetone. Over a period of 5 days, animals were freeze-substituted as follows: −90 °C for 27 h, 2 °C per hour increase for 15 h, −60 °C for 12 h, 2 °C per hour increase for 15 h, −30 °C for 32 h, 2 °C per hour increase for 17 h. At 4 °C carriers were rinsed 3 times with dry acetone for 20 min each time.

### TEM—Embedding and sectioning

Samples were infiltrated stepwise in LR-White, hard grade (London, Resin, Basingstoke, UK) and embedded in closed capsules. Polymerization was performed by UV illumination of the AFS for 24 h at 0 °C, 2 °C per hour increase for 10 h followed by 24 h at 20 °C, 2 °C per hour increase for 8 h followed by 72 h at 37 °C. Ultrathin (70 nm) sections were cut using a Leica Ultracut S ultramicrotome (Leica, Vienna, Austria) with a diamond knife (Diatome, Ltd., Biel, Switzerland) and collected on formvar-coated copper single slot grids (Agar Scientific, Stansed, UK).

### TEM—immunogold labelling

All steps of the immunolabelling were performed in a humid chamber at room temperature. Grids were floated upside down on 25 μl of aliquots of blocking solution (5% BSA, 1% Fish skin gelatin in Phosphate buffered saline (PBS)) for 30 min followed by a wash step for five times 5 min in incubation buffer (IB: 1% BSA in PBS). Incubation of primary antibodies for 120 min, Goat anti-GFP-biotin antibody, 1:300 (Rockland 600–106–215) followed by washing five times 5 min in IB. The grids were then incubated with unconjugated bridging antibodies, Rabbit anti biotin, 1:10,000 (Rockland 100–4,198) for 30 min. After washing five times 5 min in IB, the grids were incubated in Protein A gold (PAG) (10 nm, Cell Biology, Utrecht University) and washed twice, 5 min each, with IB; three times 5 min with PBS; and five times 2 min with double distilled water. Control experiments consisted of treating sections with bridging antibodies and/or PAG 10 nm alone.

After post-staining in a Leica EM AC20 (Leica, Microsystems) for 30 min in uranyl acetate at 20 °C and for 7 min in lead citrate at 20 °C sections were examined with a JEOL JEM 1010 (Jeol, Ltd, Tokyo, Japan) transmission electron microscope operating at 60 kV. Pictures were digitized using a Ditabis system (Pforzheim, Germany).

### Germ cell quantification assay

Animals for germ cell quantification assays were cultivated in an identical manner as for the germline apoptosis assay, only the staining with acridin orange was omitted. Briefly, one-day-old adult animals were used to score number of germ cells, and animals with clearly underdeveloped or abnormal looking gonads were not included in this analysis. Germ cells were scored within a fixed 900 μm^2^ square region in the late pachytene region of the germline, whereby the entire width of the germline fell within this square. Within this square, the number of germ cells in the upper half of the germline, starting from the widest point, were determined by differential interference contrast microscopy. For each genotype and condition, at least five worms per biological replicate were scored, one gonadal arm per animal was used, and at least three biological replicates were carried out.

### Western blotting

Western blotting was performed according to standard procedures. Synchronized populations were grown on NGM with induced RNAi, collected as one-day-old adults, and flash frozen in 100 μl volumes. After worm homogenization, protein content was determined using the bicinchoninic acid method. Equal amounts of total protein content were loaded on a 12.5% SDS–PAGE gel. The primary rabbit polyclonal antibodies anti-PMK-1 and anti-phospho-p38 MAPK monoclonal antibody 28B10 (Cell Signalling) were used at a 1:1,000 and 1:500 dilution, respectively, and detected with an HRP-conjugated secondary anti-rabbit antibody (No. A0545; Sigma) (1:5,000). Incubation with Supersignal West Pico chemiluminescent substrate (Pierce) generated light that was recorded on chemiluminescence film. Densitometry was performed using ImageJ software. Full scans of Western blots are presented in Supplementary Fig. 8.

The specificity of anti-PMK-1 and anti-phospho-p38 MAPK was determined by using the PMK-1 mutant *KU25 [pmk-1(km25) IV].* Three biological replicates were run.

### Statistical analysis

Statistical comparisons of fecundity and germline apoptosis levels were performed using a two-sided *t*-test, assuming unequal variance, on three or more independent biological replicates. Statistical comparisons of *in vitro* superoxide production was performed using a two-sided *t*-test, assuming unequal variance, on three or more technical replicates.

## Additional information

**Accession codes:** Crystal structure data have been deposited in the Protein Data Bank under accession code 4bja.

**How to cite this article:** De Henau, S. *et al.* A redox signalling globin is essential for reproduction in *Caenorhabditis elegans*. *Nat. Commun.* 6:8782 doi: 10.1038/ncomms9782 (2015).

## Supplementary Material

Supplementary Information Supplementary Figures 1-8 and Supplementary Tables 1-2

## Figures and Tables

**Table 1:** GLB-12 crystal structure—data collection and refinement statistics.

	**GLB-12 native**	**GLB-12 SAD**
*Data collection*		
Wavelength (Å)	0.95372	1.72200
Space group	*P*6_5_22	*P*6_5_22
Cell dimensions		
*a*, *b*, *c* (Å)	50.4, 50.4, 245.3	50.4, 50.4, 245.3
*α*, *β*, *γ* (°)	90.0, 90.0, 120.0	90.0, 90.0, 120.0
Resolution (Å)	29.85–1.65 (1.74–1.65)*	49.05–2.70 (2.85–2.70)
No. total reflections	159,514	219,339
No. unique reflections	23,429	5,773
*R*_merge_ (%)	6.4 (56.1)	6.3 (18.6)
*I*/σ(*I*)	13.9 (3.2)	49.7 (24.8)
Completeness (%)	99.8 (100)	100 (100)
Redundancy	6.8 (6.9)	38.0 (38.0)
Anomalous completeness (%)		100 (100)
Anomalous redundancy		23.0 (21.8)
		
*Refinement*		
*R*_work_/*R*_free_ (%)	17.8/23.4	
Protein residues	182 (residues 19–166, 182–215)	
Haem	1	
Water	110	
Sulfate ion	1	
Acetate	4	
B-factors (Å^2^):		
Protein	25.4	
Haem	25.7	
Water	37.4	
Sulfate ion	47.4	
Acetate	38.1	
r.m.s deviations:		
Bond lengths (Å)	0.017	
Bond angles (°)	1.6	
Ramachandran plot:		
most favoured regions (%)	92.6	
additional allowed regions (%)	7.4	

^*^Highest resolution shell is shown in parenthesis.
