# Supplementary material for: A redox signalling globin is essential for reproduction in Caenorhabditis elegans
Source: Nat Commun. 2015 Dec 1;6:8782. doi: 10.1038/ncomms9782 (PMC4686822; doi:10.1038/ncomms9782)
Supplement: Supplementary Information — Supplementary Figures 1-8 and Supplementary Tables 1-2 [file ncomms9782-s1.pdf]

Supplementary Figure 1

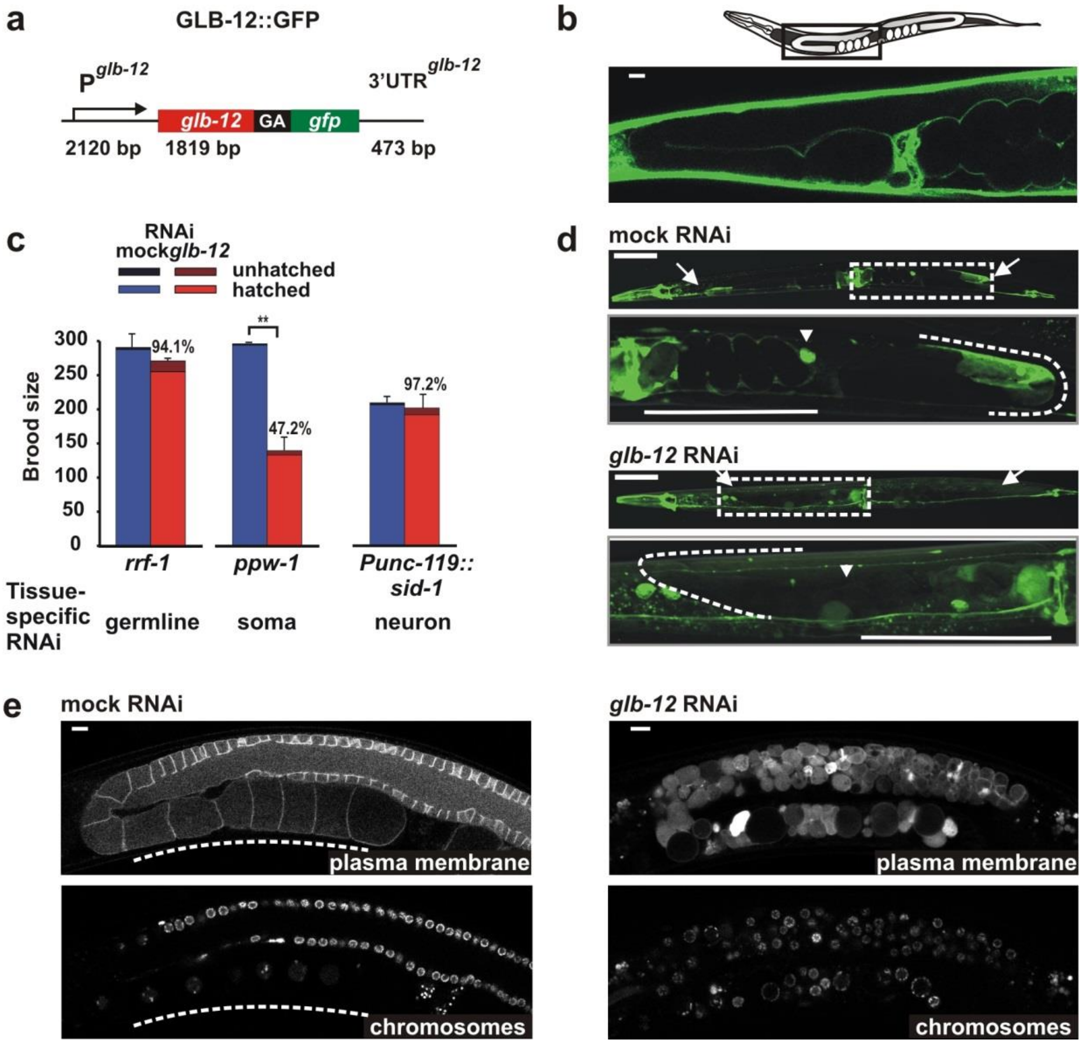

Supplementary Figure 1. GLB-12 regulates reproduction by acting from the somatic gonad. **(a)** Schematic representation of the reporter construct for *glb-12*. A GA-linker was introduced between GLB-12 and GFP. **(b)** The integrated GLB-12::GFP reporter construct does not show expression in the germline. Scale bar: 10 µm. **(c)** *glb-12* RNAi reduced fecundity in the soma-RNAi-specific strain *ppw-1*, but not in the germline-RNAi-specific strain *rrf-1* and neuronal-RNAi-specific strain *Punc-119::sid-1* (n=3). ** p<0.01 (two-sided Student *t*-test). Data are represented as mean ± SEM. **(d)** *glb-12* RNAi specifically reduced expression of the GLB-12 translational reporter in the somatic gonad. Following *glb-12* RNAi, expression was no longer seen in the distal gonadal sheath (arrow/dotted lines), the proximal part of the spermatheca (arrowhead) and the uterus (full line). Scale bar: 100 µm. **(e)** The second generation of worms exposed to of *glb-12* RNAi showed a large number of sterile animals with abnormal germline development and the absence of mature oocytes. In control worms, the region with maturing oocytes is indicated by a dashed line. Because fluorophore expression was weaker in these sterile animals, images for the two fluorophores are presented separately to improve clarity. Scale bar: 10 µm.

Supplementary Figure 2

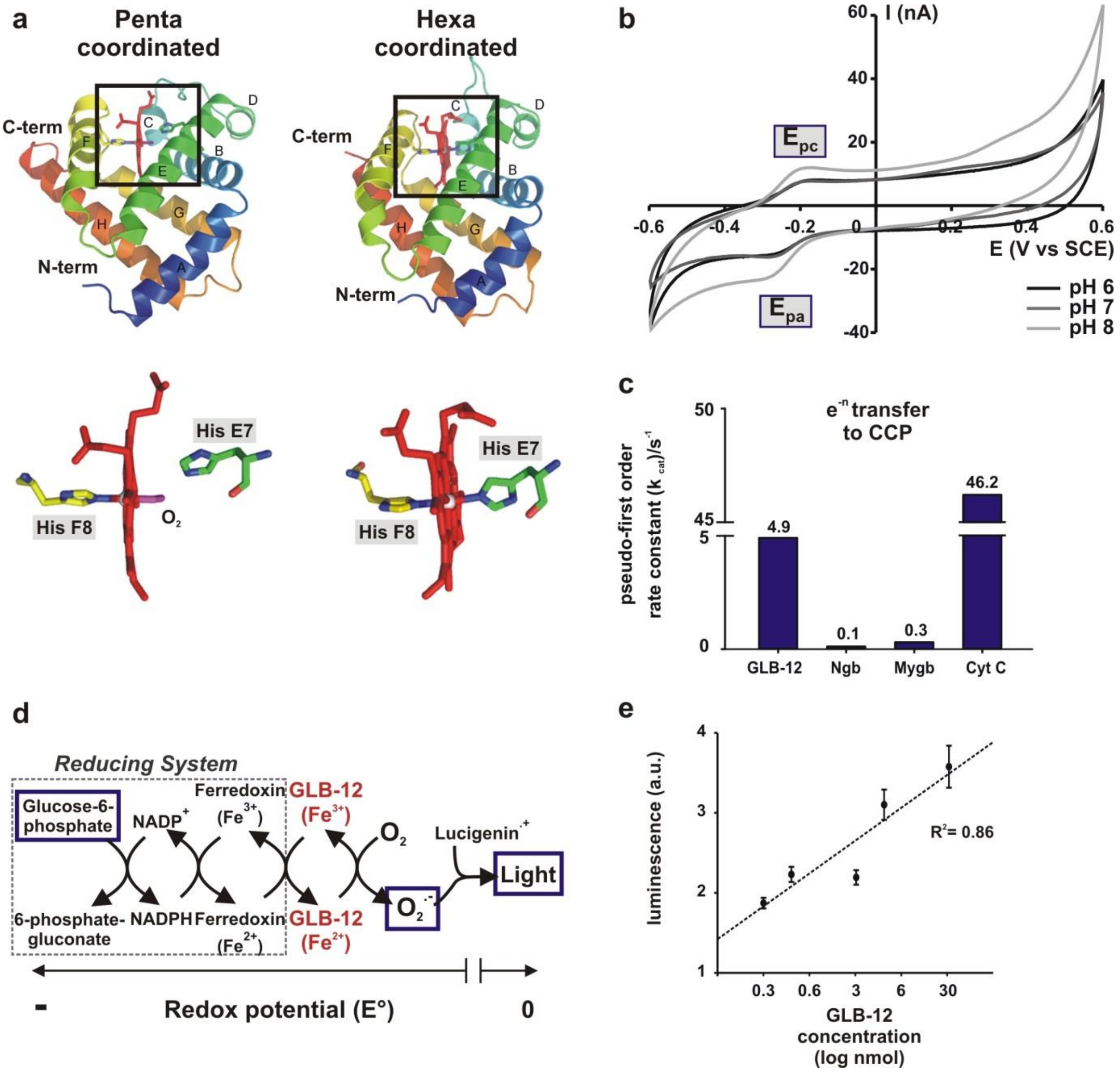

Supplementary Figure 2. GLB-12 is suited for redox chemistry and capable of $O_2^{\cdot-}$ production. **(a)** Penta- and hexacoordination in globins. In globins with a pentacoordinated heme iron, one potential binding site of the iron remains open and reversible binding of diatomic gaseous ligands is a one-step process. The oxygenated structure of sperm whale myoglobin (PDB code 1MBO) is shown as representative of a pentacoordinated globin with an exogenous ligand bound at the heme distal site. In globins with a hexacoordinated heme iron, all six coordination sites of the iron are occupied. The structure of human neuroglobin (PDB code 1OJ6) is shown as representative of a hexacoordinated globin. Ligand binding is possible, but requires the dissociation of the sixth coordination and therefore becomes more complex. In both panels the globin structures are shown in ribbon representation, with helices labeled according to the canonical globin fold. The distal HisE7 (green), the proximal HisF8 (yellow), the heme (red, with the Fe-atom in grey) and the oxygen molecule (violet) are shown in

stick representation. **(b)** The current potential behavior of a gelatin B electrode containing GLB-12, with the reduction (E_{pc}) and oxidation peak (E_{pa}), at different pH, showing that the redox couple Fe^{2+}/Fe^{3+} couple is largely pH independent within the physiological pH range. **(c)** GLB-12 showed a higher electron transfer rate to CCP compared to the hexacoordinated neuroglobin (Ngb) and pentacoordinated myoglobin (Mygb). Cytochrome C (Cyt C) is included as positive control. **(d)** The scheme shows the *in vitro* system used to measure the capacity of heme proteins to produce $O_2^{\cdot -}$. **(e)** The amount of GLB-12, expressed as log value, and luminescence increase proportionally, indicative of a 1:1 stoichiometry of $O_2^{\cdot -}$ production. Data are represented as mean ± SEM (n=2).

Supplementary Figure 3

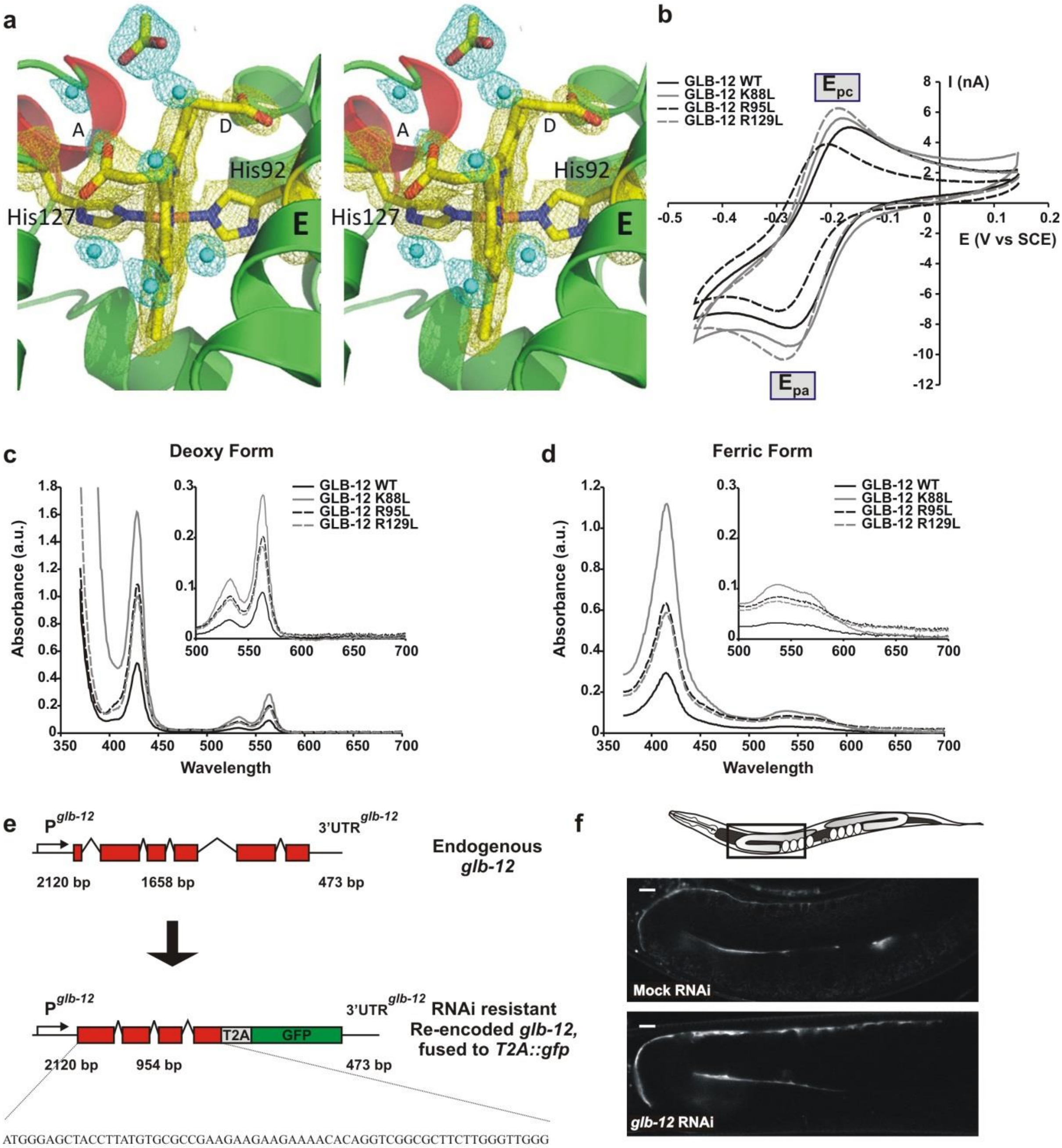

ATGGGAGCTACCTTATGTGCGCCGAAGAAGAAGAAAACACAGGTCGGCGCTTCTTGGGTTGGG
AACGAATCCGAAAACCCTTTTGATTTAGCTTTAAACAAAAAGGATCGCACACTTTTACGTGAAA
CCTGGCAGAGGCTTGATGATCCTAAAGATATTGTGGGATTAATCTTTCTTGACATTGTGAATGAC
ATCGAGCCAGATCTTAAAAAAGTGTTCGGCGTTGATAGGGCTCCTCGCGCAGCCATGTTAAAGg
taagtttaaacatatatatactaactaaccctgattatttaaattttcagATGCCTAAATTTGGTGGCCACATTTTACGCTTTTATG
AGTTTATGGAGCAATTAACTTCGATGCTCGGCACCTCGGAAAATTTAACCGGCGCCTGGCAGCT
TGTGCGCAAAACCGGCCGTTCGCATGTGCGCCAGGGTTTTCTTGAGCAGgtaagtttaaacagttcggtacta
actaaccatacatatttaaattttcagAACCAGAATCAGATGGAGAAAAACTATTTTGAGATTGTGATCAATGTTT
TTATCGAACGCCTTATCCCTTTTCTTACAGGAGAACAGGAACTTCCTTCTTCGGAAGGCAAGGA
AAATAAAAAAGTTAGGTTCGCACAGAATTACACAACCTCGCAGATAACAGACGTGTGGAAGAA
ATTTTTAAATACTGTCATTTCTCAGgtaagtttaaacatgattttactaactaactaatctgatttaaattttcagATGACCGACTC
GTTTGAACTTGAACGCGCAAAGCAGAAATCGGCTCAGACCACAAAAGCTTTAGCCCCACATCA
GCACATTGAGATTTCTGAACGCAAAAAAAAGAGGGTTGCAGAGAAACAGTCGGAAATTGAGA
ATACTGCAGTTTCGAACGAGCCGAAAGCTCAGGAACAGATGTTTGAAGATCCTTTCTAA

Supplementary Figure 3. The globin domain of GLB-12 shows structural characteristics that facilitate $O_2^{\cdot-}$ production.

(a) Stereo view of the GLB-12 heme pocket. The distal His92, the proximal His127, and the heme are shown in stick representation, together with one acetate molecule and six water molecules (cyan sphere). His92, His127 and the heme propionates (A and D) are labelled. The electron density $2F_O$-F_C map (contoured at 1 Å) is shown around the heme and the Fe-coordinated His residues (yellow mesh) and around the solvent molecules (cyan mesh). **(b)** The current potential behavior of a gelatin B electrode with WT GLB-12 and the GLB-12 mutants K88L, R95L and R129L. The reduction (E_{pc}) and oxidation peaks (E_{pa}) are indicated. The three mutations cause a small negative shift in reduction potential, from -0.226V (*vs.* SCE) for WT GLB-12, to -0.231V for GLB-12 K88L, -0.255V for GLB-12 R95L and -0.244V for GLB-12 R129L. **(c-d)** UV-Vis spectra of WT GLB-12, together with GLB-12 K88L, R95L and R129L mutant forms. The absorption spectra show that these three mutants, like WT GLB-12, are spontaneously oxidized to the ferric state (Fe^{3+}) upon exposure to air and have a hexacoordinated heme iron following reduction to a deoxy form (Fe^{2+}). **(e)** The *glb-12* gene was re-encoded to generate the RNAi-resistant *glb-12*^{RR} transgene and fused to the coding sequence for the self-cleaving peptide T2A and the *gfp* gene. The nucleotide sequence of the *glb-12*^{RR} gene is shown. **(f)** *glb-12* RNAi does not deplete the presence of GLB-12^{RR}::T2A::GFP in the somatic gonad, while this was the case for the endogenous GLB-12 fused to GFP (see also Supplementary Figure 1d). Scale bar: 100 μm.

Supplementary Figure 4

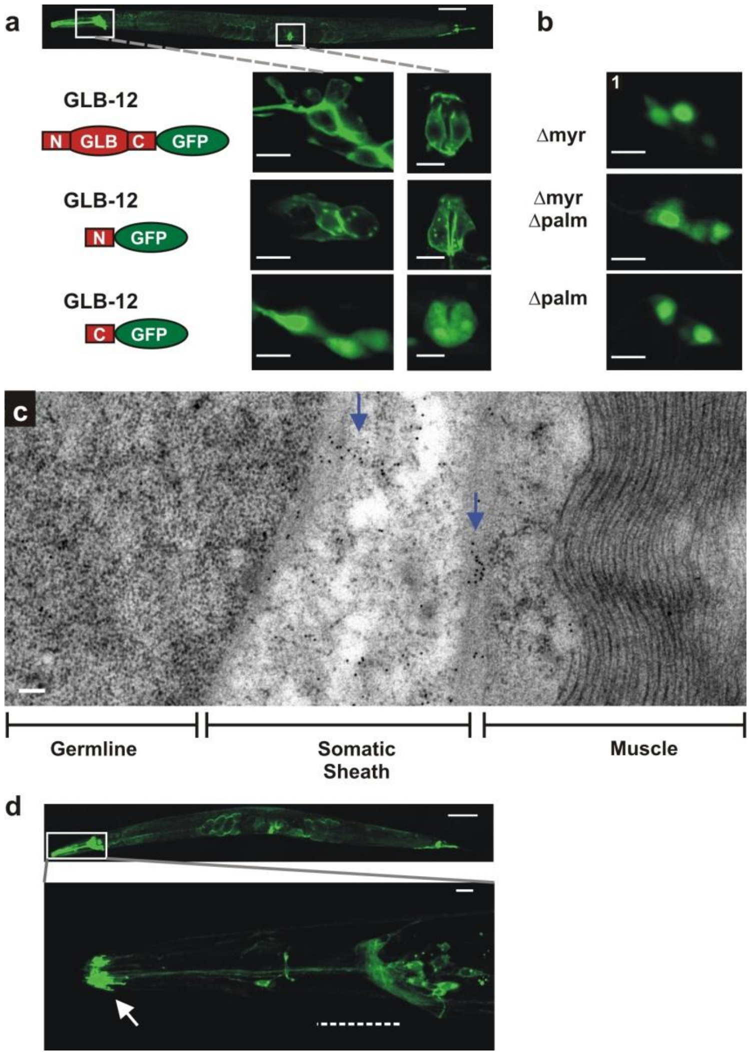

Supplementary Figure 4. GLB-12 shows a compartmentalized expression.

(a) Schematic representation of the reporters for the full-length, N- or C-terminal region of GLB-12 (scale bar: 100 µm), with a representative image of the subcellular expression of these reporters, as seen in the head neurons (middle panels) and the vulva (right panels), showing that GLB-12 is membrane-bound by its N-terminal region (scale bar: 10 µm). These tissues were chosen because of their well-defined cell body, which allows expression analysis of the GLB-12 reporters. **(b)** The subcellular expression patterns of the reporters with deleted myristoylation and/or palmitoylation sites, as seen in the head neurons, showing that also in *C. elegans* GLB-12 is membrane-bound by protein acylation. Scale bar: 10 µm **(c)** Subcellular expression pattern of GLB-12::GFP as seen by immunogold localization. Arrows indicate a clustered distribution. Scale bar: 100 nm **(d)** Subcellular expression pattern of GLB-12::GFP as seen in the head neurons, when the reporter is expressed at lower concentrations. The dotted line indicates the region of the neuronal cell bodies, the arrow points to the most anterior region of these neurons, the neuronal cilia. GLB-12::GFP accumulates at these neuronal cilia. Scale bar: 100 µm for the upper picture, 10 µm for the lower picture.

Supplementary Figure 5

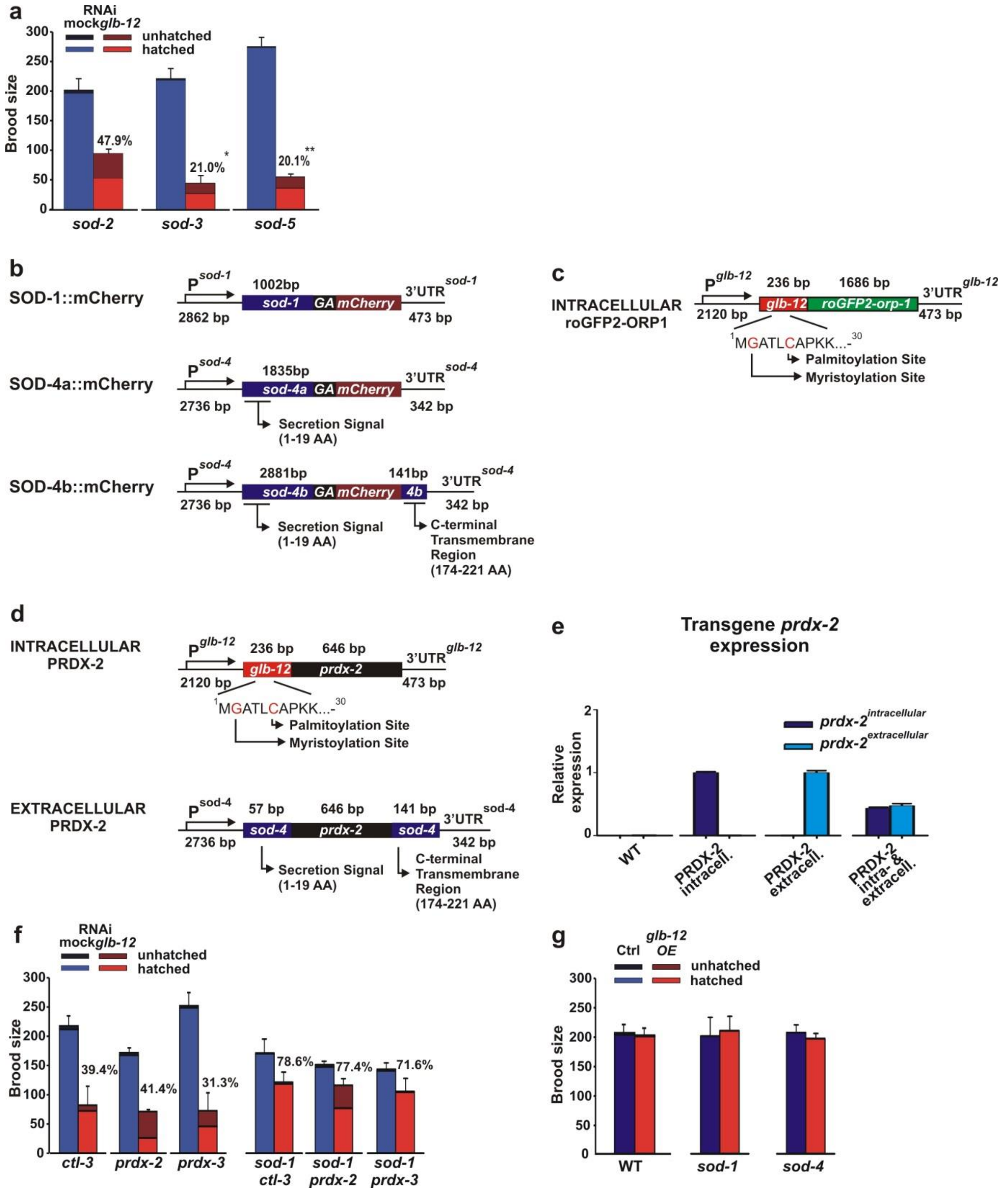

Supplementary Figure 5. GLB-12 interacts with SOD-1 and SOD-4 to produce a H_2O_2 signal.

(a) Fecundity following *glb-12* RNAi in loss-of-function mutants for SOD-2, SOD-3 and SOD-5 (n=3). Percentages show control RNAi compared to *glb-12* RNAi within a strain, * indicates significant differences compared to the WT strain. **(b)** Schematic representation of the reporter constructs for *sod-1* and the two isoforms of *sod-4*. **(c)** Schematic representation of the construct targeting roGFP2-ORP1 to the subcellular location of GLB-12. **(d)** Schematic representation of the constructs for *prdx-2* targeted to the intracellular and extracellular side of the plasma membrane. **(e)** Relative expression levels of the intracellular and extracellular *prdx-2* transgenes, determined by qPCR. **(f)** Reduction in fecundity by *glb-12* RNAi in mutants for the H_2O_2 scavengers CTL-3, PRDX-2 and PRDX-3 (n=3). **(g)** Fecundity in the WT and loss-of-function mutants for SOD-1 and SOD-4 when GLB-12 is overexpressed (n=3). *$p<0.05$; ** $p<0.01$ (two-sided Student *t*-test). All data are represented as mean ± SEM.

Supplementary Figure 6

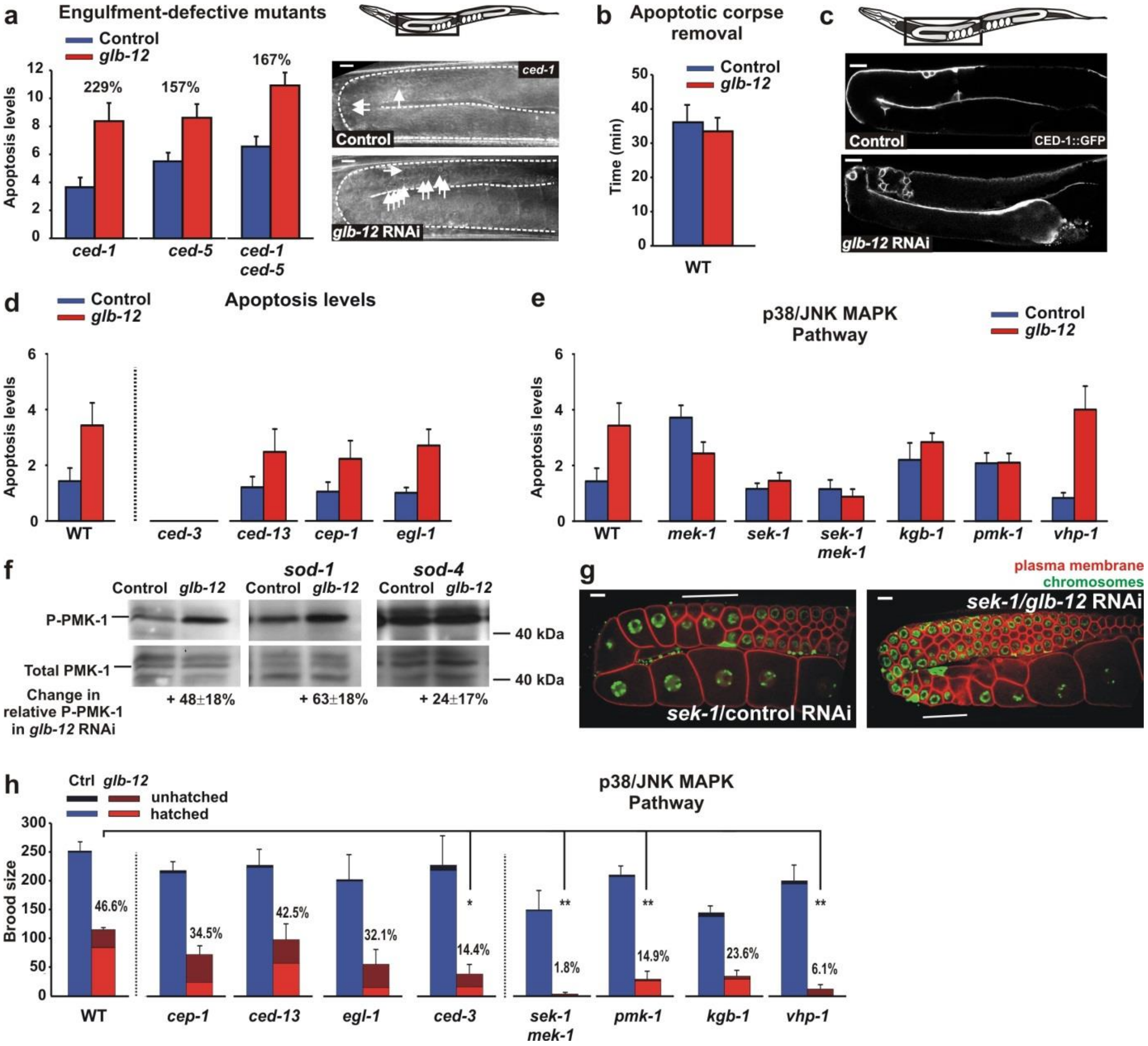

Supplementary Figure 6. GLB-12 increases germline apoptosis levels via the p38/JNK MAPK pathways.

(a) *glb-12* RNAi still caused increased germline apoptosis levels in mutants that are defective for the two parallel pathways that control cell-corpse removal (n=4). Because the dye used to score apoptosis in this study, acridin orange, will preferentially label engulfed cell corpses, it could not be used in these mutants. Therefore, apoptotic corpses in these mutants were scored by DIC. A representative image is shown for the *ced-1* mutant, with arrows indicating apoptotic cells. Scale bar: 10 µm. **(b)** No significant effect of *glb-12* RNAi was observed on the speed of cell corpse removal in WT worms. Time-lapse imaging was used to follow the presence of apoptotic corpses. (n=9 corpses for control RNAi and 13 for *glb-12* RNAi, spread over 3 replica's). **(c)** The somatic gonad does not show any obvious defects or abnormalities following *glb-12* RNAi. CED-1::GFP was used as a marker for the somatic gonad. Scale bar: 20 µm. **(d)** Absolute germline apoptosis levels following control and *glb-12* RNAi in the WT, in a CED-3 mutant and in mutants for the pro-apoptotic proteins CEP-1, CED-13

and EGL-1 (n=5). **(e)** Absolute germline apoptosis levels following control and *glb-12* RNAi in the WT and in mutants for the p38/JNK MAPK pathways (n=5). **(f)** Phosphorylated PMK-1 (P-PMK-1) levels are increased following *glb-12* RNAi in WT animals. This effect is enhanced in a *sod-1* mutant background and reduced in a *sod-4* mutant (n=3)**. (g)** Effect of *glb-12* and *sek-1* RNAi on the gonadal structure, with membranes in red and histones in green. Full line indicates the transition region pachytene - diplotene – diakenesis. Double RNAi of *glb-12* and *sek-1* still caused several gonadal defects that were also observed following *glb-12* RNAi alone, including delayed meiotic progression and abnormal germline architecture. Similar results were obtained with RNAi against *mek-1*, *pmk-1* and *kgb-1* in combination with *glb-12* RNAi. Scale bar: 10 μm. **(h)** *glb-12* RNAi caused a further decrease in fecundity in a *ced-3* mutant, in which germline apoptosis is absent, and in mutants for the p38/JNK MAPK pathways, but not in *cep-1*, *ced-13* and *egl-1* mutants (n=3). Percentages show control RNAi compared to *glb-12* RNAi within a strain. * $p<0.05$; ** $p<0.01$ (two-sided Student *t*-test). All data are represented as mean ± SEM.

Supplementary Figure 7

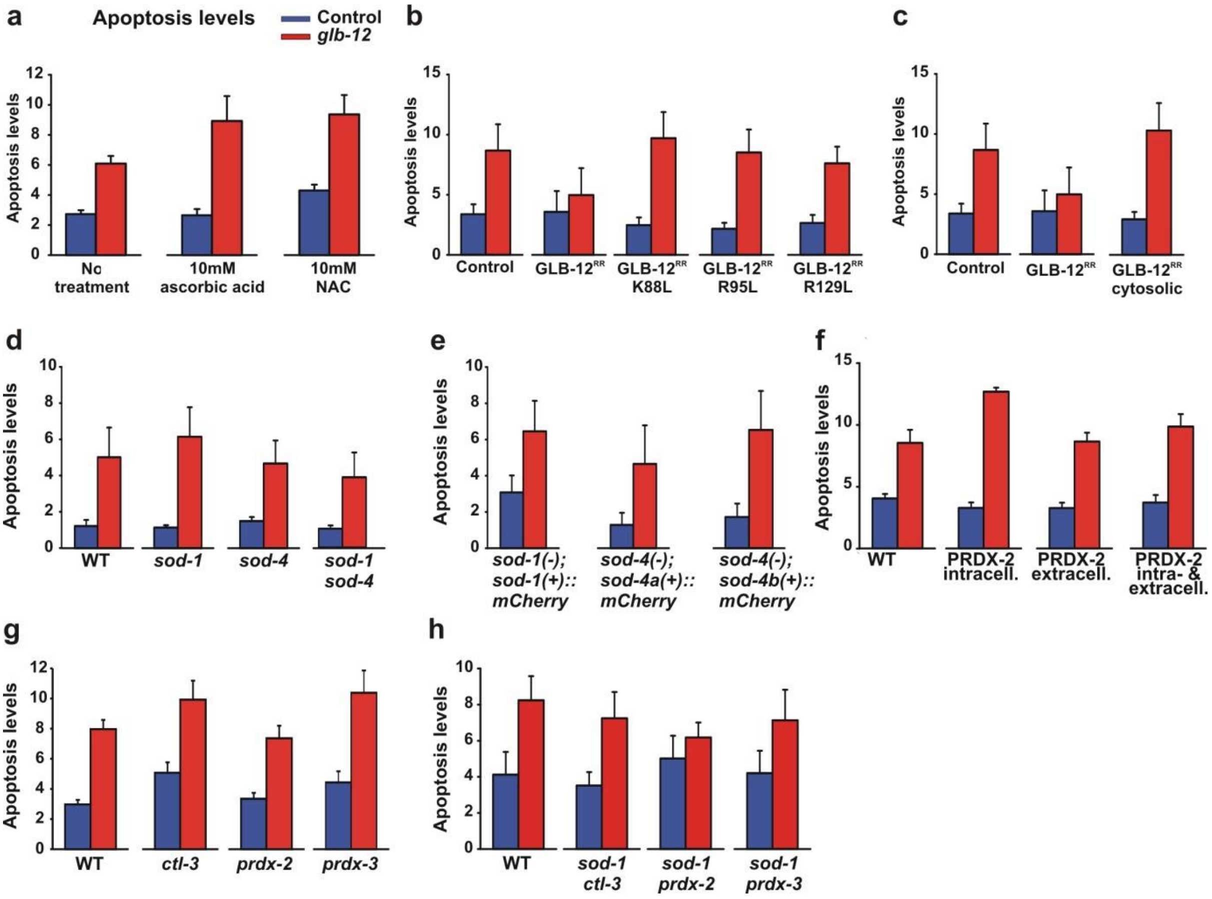

Supplementary Figure 7. GLB-12 regulates germline apoptosis levels through redox signalling. **(a)** Absolute germline apoptosis levels following control and *glb-12* RNAi in WT worms that were exposed to ascorbic acid and NAC (n=6). **(b-c)** Absolute germline apoptosis levels following control and *glb-12* RNAi in transgenic worms carrying the GLB-12^{RR} constructs (n=5). **(d)** Absolute germline apoptosis levels following control and *glb-12* RNAi in mutants for SOD-1 and SOD-4 (n=6). **(e)** Absolute germline apoptosis levels following control and *glb-12* RNAi in mutants for SOD-1 and SOD-4 that carry the SOD-1 and SOD-4 reporter constructs (n=6). **(f)** Absolute germline apoptosis levels following control and *glb-12* RNAi in transgenic worms artificially overexpressing intracellular and extracellular PRDX-2 constructs (n=5). **(g-h)** Absolute germline apoptosis levels following control and *glb-12* RNAi in mutants for the naturally occurring H_2O_2 scavengers CTL-3, PRDX-2 and PRDX-3 (n=3). All data are represented as mean ± SEM.

Supplementary Figure 8

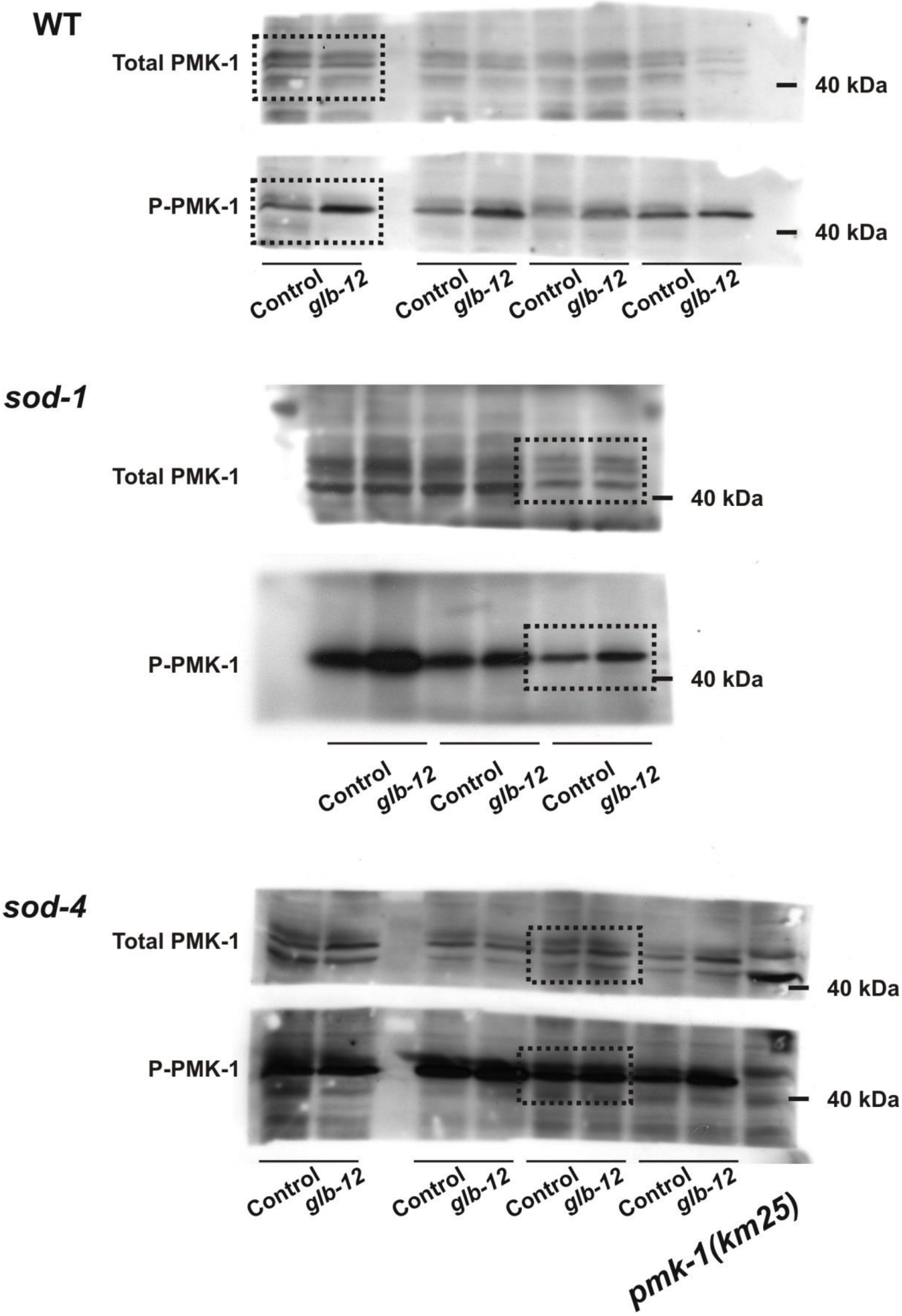

Supplementary Figure 8. Full scans of uncropped immunoblots presented in supplementary figure 6.

Dashed boxes indicate the cropped regions.

SUPPLEMENTARY TABLES

Supplementary Table 1. Germ cell quantification of *C. elegans* strains, following mock and *glb-12* RNAi.

Genotype	Control RNAi	*glb-12* RNAi	p value
WT	25.8 ± 0.9	26.7 ± 0.7	0.43
ced-13	26.9 ± 0.8	26.7 ± 1.8	0.92
egl-1	27.5 ± 1.1	26.9 ± 0.9	0.69
cep-1	25.7 ± 1.2	27.1 ± 1.9	0.57
mek-1	28.3 ± 0.7	28.7 ± 1.0	0.80
sek-1	29.5 ± 1.8	29.3 ± 1.3	0.92
mek-1 sek-1	29.2 ± 1.6	31.7 ± 2.1	0.40
kgb-1	27.5 ± 1.9	26.7 ± 1.6	0.76
pmk-1	27.9 ± 2.2	27.0 ± 2.5	0.79
vhp-1	26.4 ± 0.8	26.7 ± 1.6	0.86
WT	29.5 ± 0.5	28.6 ± 0.6	0.29
sod-1	29.9 ± 0.8	30.7 ± 1.9	0.73
sod-4	29.0 ± 1.6	30.5 ± 1.0	0.49
sod-1 sod-4	29.5 ± 0.2	29.1 ± 1.1	0.79
Control	30.3 ± 0.6	29.1 ± 1.0	0.37
glb-12^{RR}	28.9 ± 1.2	30.1 ± 1.0	0.50
glb-12^{RR} (K88L)	28.3 ± 1.0	31.0 ± 1.3	0.17
glb-12^{RR} (R95L)	29.2 ± 1.4	30.1 ± 1.0	0.65
glb-12^{RR} (R128L)	30.0 ± 1.0	29.4 ± 1.4	0.75
glb-12^{RR} cytosolic	28.8 ± 0.8	29.4 ± 1.4	0.73
Control	28.6 ± 1.0	29.9 ± 1.7	0.57
PRDX-2 extracell.	28.4 ± 1.7	29.1 ± 1.9	0.81
PRDX-2 intra-&extracell.	28.4 ± 1.3	29.7 ± 2.8	0.71
PRDX-2 intracell.	28.8 ± 0.5	31.0 ± 1.7	0.33

RNAi depletion of *glb-12* does not significantly affect the number of germ cells under the conditions used to score germline apoptosis levels (n=3) (two-sided Student *t*-test). Data shown are mean ± SEM.

Supplementary Table 2. Primer sequences used for RT-QPCR analysis and for transgene construction.

qPCR targets	Forward primer	Reverse primer
act-1 (reference gene)	gctggacgtgatcttactgattacc	gtagcagagcttctccttgatgtc
ama-1 (reference gene)	cctacgatgtatcgaggcaaa	cctccctccggtgtaataatg
cdc-42 (reference gene)	ctgctggacaggaagattacg	ctcggacattctcgaatgaag
csq-1 (reference gene)	aactgaggttctgaccgagaag	tactggtcaagctctgagtcgtc
eif-3 (reference gene)	gctgagactgttaagggaatgg	gagcgaaacagtggcataaac
pmp-3 (reference gene)	gttcccgtgttcatcactcat	acaccgtcgagaagctgtaga
prdx-2 intracellular construct	cgaatctgaaaatccatttgacttatcg	tcttgaattgtggagctggct
prdx-2 extracellular construct	tggctctctccgtttgcatt	tcagagagcgaaacatcaacga

Transgenic constructs	Forward primer	Reverse primer
glb-12 prom+gene	aaccaagatcacttttcctt	gaatggatcttcgaacatttg
glb-12 3'UTR	taaaagtatatttttatagtga	tttcaaacttgacgtcctac
glb-12 prom+gene(1-30)	aaccaagatcacttttcctt	taagtcaaatggattttcagat
glb-12 gene(210-266)	*atg*gcaaaacagaagagtgc	gaatggatcttcgaacatttg
glb-12RR gene	atgggagctaccttatgtgcgcc	gaaaggatcttcaaacatctgttcc
sod-1 prom+gene	aacagatgacaaaactggaa	ctggggagcagcgagagca
sod-1 3'UTR	tgactacctgaatcgcgtc	caaaaactggctctattcag
sod-4a prom+gene	ttgtaagagttggaacatgc	gacggtacctgtcaaacag
sod-4b prom+gene	ttgtaagagttggaacatgc	aattcctgaataatttaacg
sod-4a 3'UTR	tgatattgtctcactgtctg	ctccgaaaaagaaggttatc
sod-4b gene(174-221)+3'UTR	gtggaagaacgtattttgg	ctccgaaaaagaaggttatc
sod-4 prom+gene(1-19)	ttgtaagagttggaacatgc	ggaagcggcttcaatgcaa
gfp	ttgagtaaaggagaagaac	tttgtatagttcatccatgcc
mCherry	acaagtttgtacaaaaaagca	cttatacaattcatccatgc
roGFP2-ORP1	tctctccacctccttcaagagt	gtgatggtgatggtgatgaga
prdx-2	tcgaaagcattcatcggaaag	gtgcttcttgaagtactcttgg
unc-119	gttgctcagtaaaagaagtagaat	cttgcgaattttaacaatacttc
